# Quantitative ^1^H NMR Metabolomics Reveal Distinct Metabolic Adaptations in Human Macrophages Following Differential Activation

**DOI:** 10.3390/metabo9110248

**Published:** 2019-10-24

**Authors:** Amanda L. Fuchs, Sage M. Schiller, Wyatt J. Keegan, Mary Cloud B. Ammons, Brian Eilers, Brian Tripet, Valérie Copié

**Affiliations:** Department of Chemistry and Biochemistry, Montana State University, Bozeman, MT 59717, USA

**Keywords:** NMR, metabolomics, primary human macrophages, glycolysis, TCA cycle, oxidative stress, Kennedy pathway, immunometabolism

## Abstract

Macrophages (MΦs) are phagocytic immune cells that are found in nearly all human tissues, where they modulate innate and adaptive immune responses, thereby maintaining cellular homeostasis. MΦs display a spectrum of functional phenotypes as a result of microenvironmental and stress-induced stimuli. Evidence has emerged demonstrating that metabolism is not only crucial for the generation of energy and biomolecular precursors, but also contributes to the function and plasticity of MΦs. Here, 1D ^1^H NMR-based metabolomics was employed to identify metabolic pathways that are differentially modulated following primary human monocyte-derived MΦ activation with pro-inflammatory (M1) or anti-inflammatory (M2a) stimuli relative to resting (M0) MΦs. The metabolic profiling of M1 MΦs indicated a substantial increase in oxidative stress as well as a decrease in mitochondrial respiration. These metabolic profiles also provide compelling evidence that M1 MΦs divert metabolites from *de novo* glycerophospholipid synthesis to inhibit oxidative phosphorylation. Furthermore, glycolysis and lactic acid fermentation were significantly increased in both M1 and M2a MΦs. These metabolic patterns highlight robust metabolic activation markers of MΦ phenotypes. Overall, our study generates additional support to previous observations, presents novel findings regarding the metabolic modulation of human MΦs following activation, and contributes new knowledge to the rapidly evolving field of immunometabolism.

## 1. Introduction

The rapidly expanding field of immunometabolism converges on the cross-talk between metabolism and immune cell function, with recent findings bringing this area of research to the forefront of immunology [1,2]. Metabolomics research aims to elucidate cellular and/or systemic perturbations in biochemical pathways by identifying and quantifying changes in small molecule metabolite profiles within complex biological systems [3,4]. Notably, metabolites, such as succinate and itaconate, have been shown to function as signaling molecules and to mediate macrophage (MΦ) cellular phenotypes, in addition to providing metabolic precursors [5,6]. Studies have indicated that pro- and anti-inflammatory MΦs employ markedly different metabolic strategies with regard to central carbon metabolic pathways, including glycolysis, oxidative phosphorylation, and fatty acid metabolism, reinforcing the concept that immunometabolism is critical to immune cell functions [7,8,9].

Pro-inflammatory, or classically activated, (M1) MΦs play a vital role in host defense through the clearance of bacteria, foreign particles, and cellular debris [10,11]. However, aberrant M1 MΦ function has been implicated in several pathologies, including type 2 diabetes, atherosclerosis, colitis, and Crohn’s disease [12,13,14,15]. Succinate has been discovered to be an inflammatory regulator and metabolic marker of M1 MΦs, which is due in part to its concentration-dependent effect on the inhibition of prolyl hydroxylases, the stabilization of hypoxia-inducible factor 1α (HIF-1α), the upregulation of pro-inflammatory cytokines, such as interleukin-1β (IL-1β), and the upregulation of pro-inflammatory gene expression [5,6]. Furthermore, stabilized HIF-1α has been shown to bind HIF-1β, which is a constitutively expressed protein subunit that initiates the expression of additional target genes, including those encoding glycolytic enzymes and glucose transporters [16,17]. An increased flow of intermediates through glycolysis has been reported to be a characteristic metabolic signature of M1 MΦ activation, and previous studies have demonstrated that a decreased flow of metabolites through glycolysis downregulates M1 MΦ effector functions, including phagocytosis, the capacity to generate reactive oxygen species (ROS), and the ability to generate pro-inflammatory cytokines [18,19,20].

Anti-inflammatory, or alternatively activated, (M2) MΦs clear apoptotic cells, promote and regulate wound healing, and alleviate inflammatory responses [10,21]. Although M2 MΦs are more favorably viewed than their M1 MΦ counterparts due to their anti-inflammatory characteristics, they have been implicated in several pathologies, including tumorigenesis, T_H_2-driven allergic inflammation, and idiopathic pulmonary fibrosis [10]. Numerous M2 MΦ subtypes, associated with distinct functional phenotypes, have also been identified, including M2a, M2b, M2c, and M2d [21]. However, M2a MΦs, which are activated using interleukin-4 (IL-4) stimuli, are presently the best-characterized M2 phenotype [21]. Disparate from M1 MΦs, M2a MΦ activation leads to a metabolic switch from glycolysis to oxidative phosphorylation and fatty acid beta oxidation for adenosine triphosphate (ATP) production and energetic homeostasis [1,7]. Using ^14^C-labeled glucose and oleic acid stimulation, Vats et al. demonstrated that M2 MΦs lower their glucose consumption, and increase fatty acid uptake and beta oxidation to fuel mitochondrial oxidative phosphorylation, in addition to enhancing the expression of fatty acid oxidation genes, relative to M1 MΦs [7]. Moreover, Huang et al. determined that lysosomal acid lipase (LAL)-mediated lipolysis is crucial for the generation of M2 MΦ activation hallmarks, including the promotion of oxidative phosphorylation and increased respiratory capacity [22].

While previous studies have identified significant metabolic differences between M1 and M2 MΦs, the overwhelming majority of these studies have been conducted using bone marrow-derived murine MΦs and immortalized murine and human cell lines [17], which limit their relevance to human health. Several inquiries have indeed highlighted notable distinctions between human and murine MΦs, including significant differences in cell surface markers, nitric oxide (NO) production, NO synthase transcript expression, arginine metabolism, and transcriptomic and proteomic profiles [10,23,24,25]. In particular, metabolic enzymes involved in arginine metabolism, such as arginase 1 (Arg1) and inducible NO synthase (iNOS), are expressed in murine MΦs; however, comparable expression has not been observed in *in vitro* cultures of M1 or M2a human MΦs [10,24]. In addition, although primary human cells present more physiologically accurate phenotypic and metabolic characteristics relevant to *in vivo* cellular environments, the usage of such cell cultures in metabolomics studies is currently limited [26]. To further understand and appreciate the potential biochemical discrepancies and functional differences between murine and human MΦ responses, additional research is needed to clearly establish the extent of human MΦ phenotypic heterogeneity, with a particular emphasis on primary human MΦs.

In this study, we sought to elucidate the metabolic consequences of pro-inflammatory and anti-inflammatory stimuli on primary human monocyte-derived MΦs, using CD14^+^ magnetic-activated cell sorting (MACS) technology, *in vitro* MΦ differentiation and activation schemes, 1D ^1^H NMR metabolomics, metabolite profiling using Chenomx NMR Suite software, and multivariate statistical analysis. To generate M1 and M2a MΦs, naïve cells were stimulated with MΦ colony-stimulating factor (M-CSF) and a combination of lipopolysaccharide (LPS) and interferon-γ (IFN-γ) or IL-4, respectively, for 72 hrs, whereas resting M0 MΦs were generated using M-CSF with no additional stimuli. Subsequently, intra- and extracellular metabolites were extracted, followed by 1D ^1^H NMR acquisition and spectral profiling of resulting metabolite mixtures. Results from this study highlight major metabolic pathways that are differentially modulated in activated human MΦs, including glycolysis, lactic acid fermentation, the tricarboxylic acid (TCA) cycle, glutathione metabolism, oxidative stress, and *de novo* glycerophospholipid synthesis within the Kennedy pathway. The functional significance of the observed metabotypes (i.e., resulting metabolite profiles) found to be associated with M1 and M2a MΦ cellular phenotypes is also discussed.

## 2. Results

### 2.1. Quantitative Metabolic Profiles Differentiate between MΦ Activation States

To identify characteristic metabolic patterns associated with MΦ activation phenotypes, metabolite profiles of M0, M1, and M2a MΦs cultured *in vitro* were characterized using an untargeted ^1^H NMR metabolomics approach. One-dimensional (1D) ^1^H NMR spectra of intra- and extracellular MΦ metabolite extracts (Figure S1) were recorded on Montana State University (MSU)’s 600 MHz (^1^H Larmor frequency) solution NMR spectrometer. This approach facilitated the deconvolution of complex NMR spectral patterns [27] and the identification and quantitation of 51 metabolites in these cell cultures.

**Figure 1:**
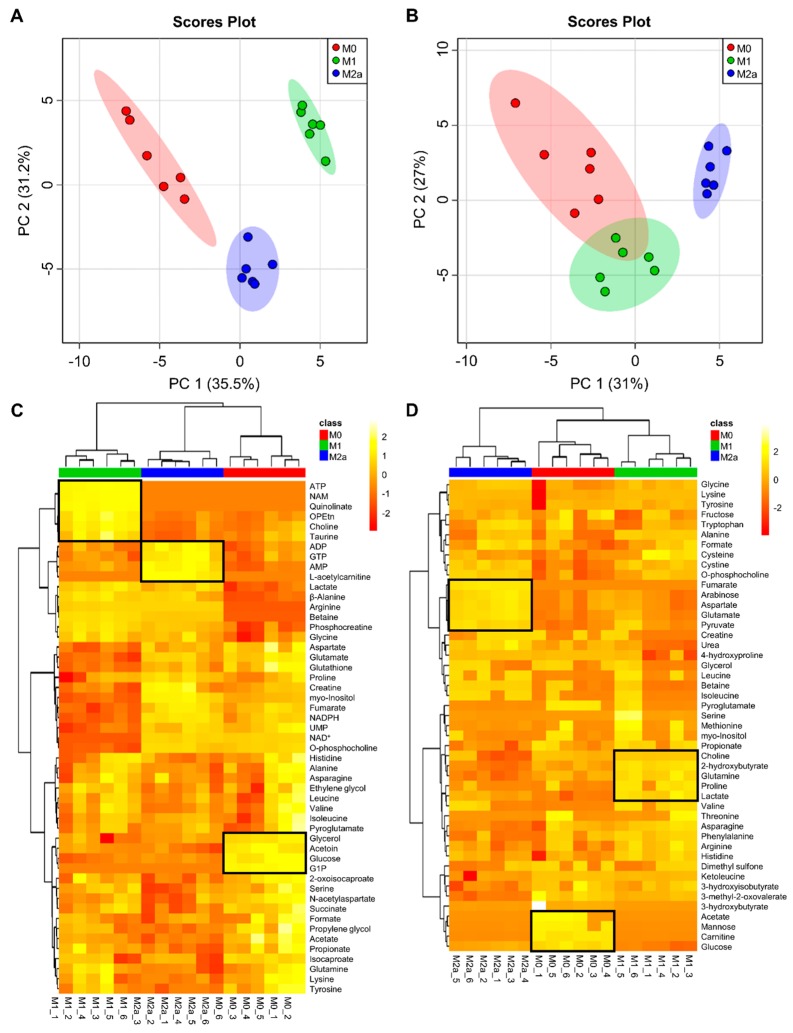
Multivariate statistical analysis reveals metabolic differences between macrophage (MΦ) activation states. Two-dimensional principal component analysis (2D-PCA) scores plots generated by analysis of metabolic profiles from intra- (**A**) and extracellular (**B**) MΦ metabolite extracts (M0, red; M1, green; M2a, blue), with shaded regions illustrating respective 95% confidence intervals. Hierarchical clustering analysis (HCA) and heatmap visualization of metabolic profiles from intra- (**C**) and extracellular (**D**) MΦ metabolite extracts were performed using a Euclidean distance calculated from metabolite abundance and a Ward clustering algorithm. The upmost column bar is colored according to the MΦ activation state (M0, red; M1, green; M2a, blue), and the color scale represents the scaled abundance of each metabolite, with yellow indicating high abundance and red indicating low abundance. Particular sets of discriminatory metabolites are highlighted in boxed regions on the heatmaps. Abbreviations denote: ADP, adenosine diphosphate; AMP, adenosine monophosphate; ATP, adenosine triphosphate; G1P, glucose-1 phosphate; GTP, guanosine triphosphate; NAD^+^, nicotinamide adenine dinucleotide; NADPH, reduced nicotinamide adenine dinucleotide phosphate; NAM, niacinamide; OPEtn, o-phosphoethanolamine; UMP, uridine monophosphate.

Two-dimensional principal component analysis (2D-PCA) scores plots of intra- and extracellular metabolite profiles (Figure 1A,B, respectively; see Figure S2 for corresponding PCA loadings plots; see File S1 and File S2 for corresponding PCA loadings) demonstrated that M0, M1, and M2a MΦs are metabolically distinct from one another, with the most striking separation observed between M0 and M1 MΦs along the principal component 1 (PC1) dimension of the 2D-PCA scores plot (Figure 1A) of the intracellular metabolite profiles. The intra- and extracellular metabolite datasets were subjected to hierarchical clustering analysis (HCA) and heatmap generation to visually assess which metabolites contributed the most significantly to the discrimination between MΦ activation states (Figure 1C,D, respectively). The intracellular metabolite profiles of each MΦ activation state presented a unique metabolic signature that was not observed in the other activation states. These included increased concentrations of metabolites such as ATP, niacinamide, quinolinate, phosphoethanolamine, choline, and taurine in M1 MΦs, adenosine diphosphate (ADP), guanosine triphosphate (GTP), adenosine monophosphate (AMP), and L-acetylcarnitine in M2a MΦs, and glycerol, acetoin, glucose, and glucose-1-phosphate in M0 MΦs, as indicated in Figure 1C. MΦ activation states were also distinguishable based upon their extracellular metabolite profiles, with increased concentrations of metabolites such as choline, 2-hydroxybutyrate, glutamine, proline, and lactate in the extracellular milieu of M1 MΦs, fumarate, arabinose, aspartate, glutamate, and pyruvate in M2a MΦs, and acetate, mannose, carnitine, and glucose in M0 MΦs, as indicated in Figure 1D.

### 2.2. Glycolytic Activity and Lactic Acid Fermentation Are Universal Markers of Activation

**Figure 2:**
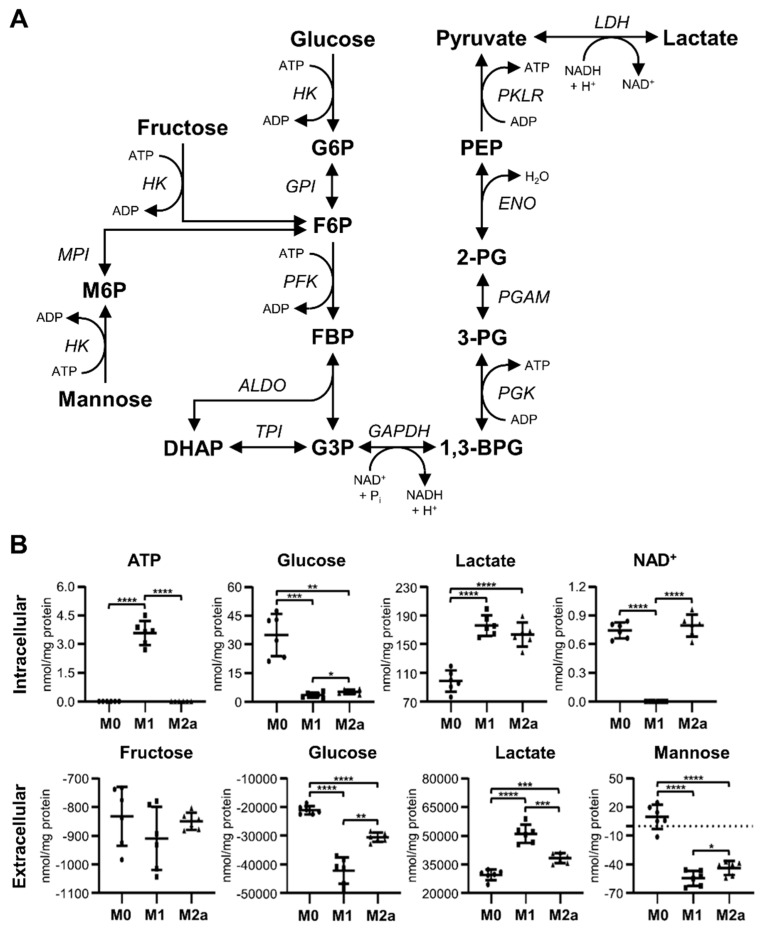
Increased glycolytic activity and lactate production are universal markers of MΦ activation. Schematic diagram of glycolysis and lactic acid fermentation pathways (**A**), and quantitative levels of corresponding metabolites (**B**) detected in intra- and extracellular MΦ metabolite extracts (mean ± SD). Unpaired parametric *t*-tests with Welch’s correction (two-tailed; *p < 0.05; **p < 0.01; ***p < 0.001; ****p < 0.0001). Abbreviations denote: 2-PG, 2-phosphoglycerate; 3-PG, 3-phosphoglycerate; ADP, adenosine diphosphate; *ALDO*, aldolase; ATP, adenosine triphosphate; BPG, bisphosphoglycerate; DHAP, dihydroxyacetone phosphate; *ENO*, enolase; F6P, fructose 6-phosphate; FBP, fructose bisphosphate; G3P, glyceraldehyde 3-phosphate; G6P, glucose 6-phosphate; *GAPDH*, glyceraldehyde-3 phosphate dehydrogenase; *GPI*, glucose-6 phosphate isomerase; *HK*, hexokinase; *LDH*, lactate dehydrogenase; M6P, mannose 6-phosphate; NAD^+^, nicotinamide adenine dinucleotide; NADH, reduced nicotinamide adenine dinucleotide; PEP, phosphoenolpyruvate; *PFK*, phosphofructokinase; *PGAM*, phosphoglycerate mutase; *PGK*, phosphoglycerate kinase; *PKLR*, pyruvate kinase L/R; *TPI*, triosephosphate isomerase.

The metabolic profiling of intra- and extracellular MΦ metabolite extracts revealed that M1 and M2a MΦ activation states induced significant metabolic changes, especially with regard to glycolytic and lactic acid fermentation pathways (Figure 2A), when compared to the metabolite profiles of M0 MΦs. M1 and M2a MΦs exhibited intracellular glucose levels that were –9.85 and –6.68-fold lower, respectively, and intracellular lactate levels that were 1.79 and 1.66-fold higher, respectively, relative to those found in M0 MΦs (Figure 2B and Table 1). These patterns were also reflected in the extracellular metabolite profiles, where M0, M1, and M2a MΦs displayed an average deficit of –21.04, –42.21, and –30.55 µmol glucose/mg protein, and an average surplus of 29.47, 51.05, and 38.15 µmol lactate/mg protein, respectively (Figure 2B and Table 2), relative to sham extracellular metabolite extract controls. Distinct metabolic characteristics of M1 MΦs included a significant, 9.14-fold, increase in intracellular ATP levels, and a significant –3.79-fold decrease in intracellular nicotinamide adenine dinucleotide (NAD^+^) levels (Figure 2B and Table 1), relative to M0 MΦs. No significant changes were observed with respect to extracellular fructose concentrations. However, extracellular mannose concentrations were found to be significantly decreased in M1 and M2a MΦs, with M0, M1, and M2a MΦs exhibiting average concentrations of 9.84, –54.62, and –43.79 nmol mannose/mg protein, respectively (Figure 2B and Table 2), relative to sham extracellular extract controls.

Significant differences in metabolite profile patterns were also established for M1 relative to M2a MΦs. These included a 9.14-fold increase in intracellular ATP, and –1.47 and –4.06-fold decreases in intracellular glucose and NAD^+^ levels, respectively (Figure 2B and Table S2). In addition, the extracellular metabolite profiles of M1 MΦs exhibited significant increases in lactate and decreases in glucose and mannose, relative to the extracellular metabolite profiles of M2a MΦs (Figure 2B and Table 2). These results support the notion that elevated glycolytic and lactic acid fermentation activities are robust markers of MΦ activation, with M1 MΦs displaying significantly greater metabolic activity with respect to these specific pathways compared to M2a MΦs.

### 2.3. M1 and M2a MΦs Exhibit Distinct Anaplerotic Trends Corresponding to the TCA Cycle

**Figure 3:**
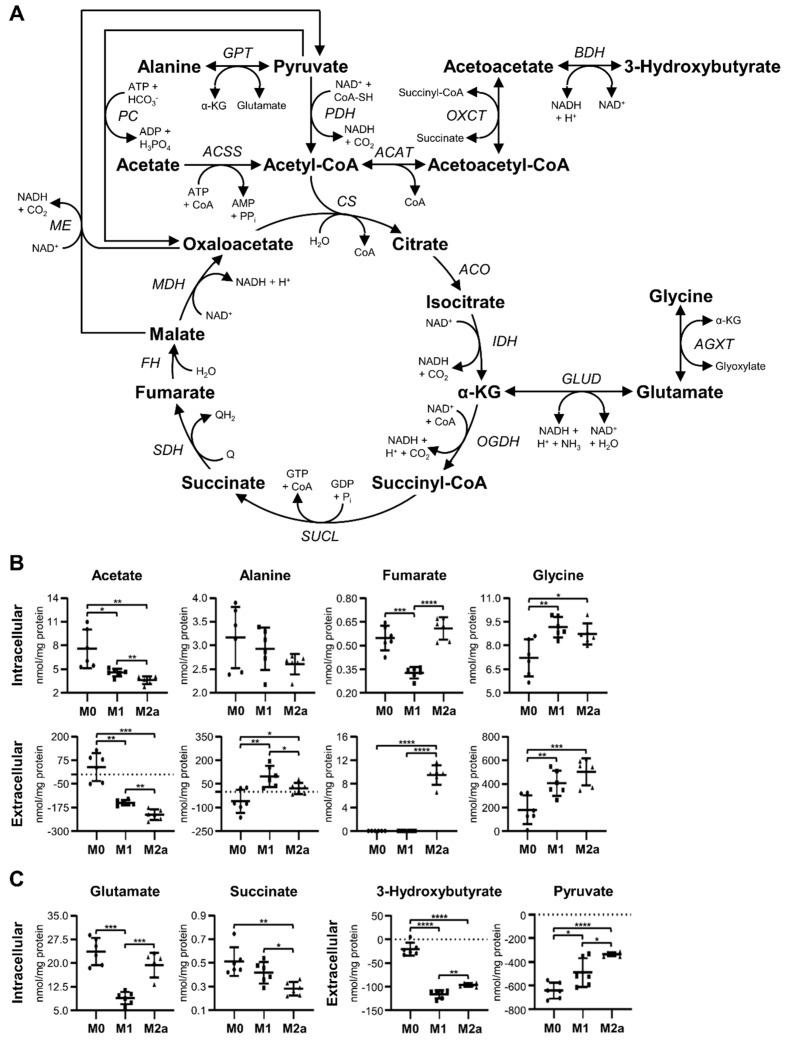
M1 and M2a MΦs exhibit contrasting substrate utilization strategies regarding the tricarboxylic acid (TCA) cycle. Schematic diagram of metabolite flow through the TCA cycle (**A**), and quantitative levels of corresponding metabolites (**B,C**) detected in intra- and extracellular MΦ metabolite extracts (mean ± SD). Unpaired parametric *t*-tests with Welch’s correction (two-tailed; *p < 0.05; **p < 0.01; ***p < 0.001; ****p < 0.0001). Abbreviations denote: *ACAT*, acetyl-CoA acetyltransferase; *ACO*, aconitase; *ACSS*, acetyl-CoA synthetase; *AGXT*, alanine-glyoxylate transaminase; AMP, adenosine monophosphate; ATP, adenosine triphosphate; *BDH*, 3-hydroxybutyrate dehydrogenase; CoA, coenzyme A; *CS*, citrate synthase; *FH*, fumarate hydratase; GABA, γ-aminobutyrate; GDP, guanosine diphosphate; *GLUD*, glutamate dehydrogenase; *GPT*, glutamate pyruvate transaminase; GTP, guanosine triphosphate; *IDH*, isocitrate dehydrogenase; *MDH*, malate dehydrogenase; *ME*, malic enzyme; NAD^+^, nicotinamide adenine dinucleotide; NADH, reduced nicotinamide adenine dinucleotide; *OGDH*, oxoglutarate dehydrogenase; *OXCT*, 3-oxoacid CoA-transferase; *PC*, pyruvate carboxylase; *PDH*, pyruvate dehydrogenase; Q, quinone; QH_2_, quinol; *SDH*, succinate dehydrogenase; *SUCL*, succinate-CoA ligase; α-KG, α-ketoglutarate.

M1 and M2a MΦs presented significantly different metabolic trends with respect to TCA cycle intermediates and substrates (Figure 3A), relative to M0 MΦs. Initial analyses focused on metabolites that can enter the TCA cycle at the acetyl-coenzyme A (acetyl-CoA) entry point, including acetate, alanine, 3-hydroxybutyrate, and pyruvate (Figure 3A). Intracellular acetate was significantly decreased in M1 and M2a MΦs by –1.66 and –2.11-fold, respectively, relative to M0 MΦs (Figure 3B and Table 1). This trend was also reflected in the extracellular metabolite profiles, where the spent medium of M0, M1, and M2a MΦs contained average concentrations of 39.73, –150.67, and –213.14 nmol acetate/mg protein, respectively (Figure 3B and Table 2), relative to sham extracellular extract controls. In addition, M1 MΦs displayed a 1.27-fold increase in intracellular acetate and significantly elevated concentrations of extracellular acetate relative to M2a MΦs (Figure 3B, Table 2, and Table S2). No significant changes were observed with respect to intracellular alanine levels. However, extracellular alanine levels were significantly increased in M1 and M2a MΦs compared to M0 MΦs, with spent culture medium of M0, M1, and M2a MΦs possessing average concentrations of –60.54, 98.11, and 20.73 nmol alanine/mg protein, respectively (Figure 3B and Table 2), relative to sham extracellular extract controls. Furthermore, M1 MΦs secreted a significantly greater amount of alanine into the media relative to M2a MΦs (Figure 3B and Table 2). Extracellular 3-hydroxybutyrate levels were significantly decreased in M1 and M2a MΦs relative to M0 MΦs, with M0, M1, and M2a MΦs exhibiting average deficits of –20.75, –116.64, and –96.50 nmol 3-hydroxybutyrate/mg protein, respectively (Figure 3C and Table 2), relative to sham extracellular extract controls. M1 and M2a MΦs also had significantly increased levels of extracellular pyruvate relative to M0 MΦs, with M0, M1, and M2a MΦs displaying average deficits of –642.79, –489.93, and –336.50 nmol pyruvate/mg protein, respectively (Figure 3C and Table 2), relative to sham extracellular extract controls. However, M1 MΦs consumed significantly greater amounts of 3-hydroxybutyrate and pyruvate from the media compared to M2a MΦs (Figure 3B and Table 2). Altogether, these results suggest that M1 and M2a MΦs preferentially utilize acetate and 3-hydroxybutyrate as their extracellular carbon sources, compared to utilizing pyruvate, relative to M0 MΦs. Moreover, M1 MΦs utilize 3-hydroxybutyrate to a significantly greater extent than M2a MΦs, and secrete greater amounts of alanine into the cell culture medium than M0 and M2a MΦs, which is potentially due to the preferred conversion of pyruvate to alanine.

These data are consistent with published reports on the differential O_2_ consumption of MΦ subtypes. For example, O_2_ consumption has been shown to decrease in M1-activated MΦs [5,6], which is consistent with our MΦ metabolic profiling data that suggest that our M1 MΦs are consuming 3-hydroxybutyrate from the extracellular cell culture media without the need to invoke the *de novo* synthesis of 3-hydroxybutyrate from ketogenic pathways.

Subsequent analyses were performed on metabolites that serve as TCA cycle intermediates and which can participate in anaplerotic reactions to replenish critical intermediates of the TCA cycle. Such metabolites included fumarate, glycine, glutamate, and succinate (Figure 3A). M1 MΦs demonstrated significantly decreased levels of intracellular fumarate, –1.68 and –1.86-fold, relative to M0 and M2a MΦs, respectively (Figure 3B, Table 1, and Table S2), whereas extracellular fumarate was only detected in M2a MΦs, at a level of 9.51 nmol fumarate/mg protein (Figure 3B and Table 2). M1 and M2a MΦ metabolite profiles revealed significantly increased levels of intracellular glycine, 1.27 and 1.21-fold, relative to M0 MΦs, respectively (Figure 3B and Table 1). This trend is also distinct from the one observed for extracellular glycine levels, whereby M0, M1, and M2a MΦ cultures contained average concentrations of 179.01, 404.88, and 501.91 nmol glycine/mg protein in their spent media, respectively (Figure 3B and Table 2), relative to sham extracellular extract controls. Intracellular levels of glutamate were significantly decreased in M1 MΦs, amounting to –2.67 and –2.19-fold decreases, relative to M0 and M2a MΦs, respectively (Figure 3C, Table 1, and Table S2). In addition, M2a MΦs displayed –1.82 and –1.48-fold decreased levels of intracellular succinate relative to M0 and M1 MΦs, respectively (Figure 3C, Table 1, and Table S2). These findings support the notion that M1 MΦ activation leads to an impaired conversion of succinate to fumarate and to an increased requirement for metabolic reactions associated with the generation of α-ketoglutarate (α-KG), such as those catalyzed by glutamate dehydrogenase (GLUD), glutamate-pyruvate transaminase (GPT), or alanine-glyoxylate transaminase (AGXT; Figure 3A) enzymes.

### 2.4. Activated MΦs Undergo Significant Oxidative Stress

**Figure 4:**
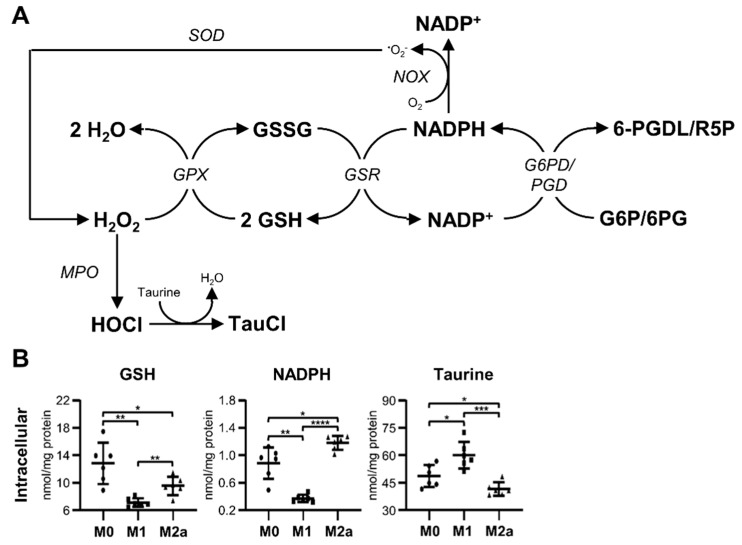
Activation induces significant oxidative stress in MΦs. Schematic diagram of glutathione metabolism and oxidative stress pathways (**A**), and quantitative levels of corresponding metabolites (**B**) detected in intracellular MΦ metabolite extracts (mean ± SD). Unpaired parametric *t*-tests with Welch’s correction (two-tailed; *p < 0.05; **p < 0.01; ***p < 0.001; ****p < 0.0001). Abbreviations denote: GSH, reduced glutathione; GSSG, oxidized glutathione; NADP^+^, nicotinamide adenine dinucleotide phosphate; NADPH, reduced nicotinamide adenine dinucleotide phosphate; G6P, glucose 6-phosphate; *G6PD*, glucose-6-phosphate dehydrogenase; *GPX*, glutathione peroxidase; *GSR*, glutathione-disulfide reductase; *MPO*, myeloperoxidase; *NOX*, NADPH oxidase; 6PG, 6-phosphogluconate; *PGD*, phosphogluconate dehydrogenase; 6-PGDL, 6-phosphoglucono-1,5-lactone; R5P, ribulose 5-phosphate; *SOD*, superoxide dismutase; TauCl, taurine chloramine.

MΦ activation resulted in intracellular metabolic adaptations associated with glutathione metabolism and pathways involved in mitigating oxidative stress (Figure 4A), which were significantly different from the metabolic pathways used preferentially by M0 MΦs. The intracellular metabolite profiles of M1 and M2a MΦs featured significantly decreased levels of reduced glutathione (GSH), –1.81 and –1.34-fold, respectively, relative to M0 MΦs (Figure 4B and Table 1). In addition, M1 MΦs contained significantly less intracellular GSH, –1.34-fold, compared to M2a MΦs (Figure 4B and Table S2). Reduced nicotinamide adenine dinucleotide phosphate (NADPH) was depleted in M1 MΦs relative to M0 and M2a MΦs, amounting to –2.40 and –3.20-fold decreases, respectively (Figure 4B, Table 1, and Table S2). On the other hand, M2a MΦs contained significantly more NADPH, 1.34-fold, compared to M0 MΦs (Figure 4B and Table 1). M1 MΦs also contained significantly increased levels of intracellular taurine relative to M0 and M2a MΦs, amounting to 1.23 and 1.45-fold increases, respectively (Figure 4B, Table 1, and Table S2), while M2a MΦs had significantly less intracellular taurine, a –1.18-fold decrease, compared to M0 MΦs (Figure 4B and Table 1). Collectively, these results demonstrate that MΦ activation induces oxidative stress in both M1 and M2a MΦs. Furthermore, M1 MΦs appear to consume NADPH to a greater extent than M0 or M2a MΦs, possibly to generate substantial levels of reactive oxygen species (ROS) as part of the M1 MΦ immune phenotype. Increased levels of taurine in M1 MΦs may also be used to compensate for or to neutralize excessive or harmful ROS production.

### 2.5. M1 MΦs Manipulate the Kennedy Pathway

**Figure 5:**
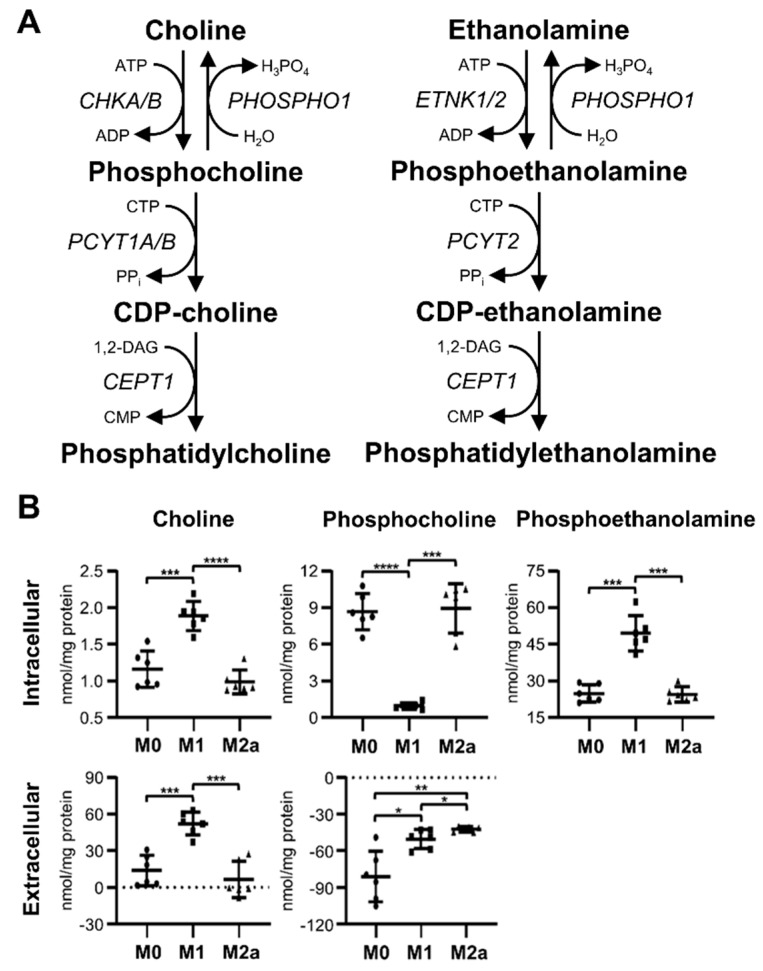
M1 MΦs have a distinct metabolic signature characterized by alterations of metabolite flow within the Kennedy pathway. Schematic diagram of metabolite flow through the Kennedy pathway (**A**), and quantitative levels of corresponding metabolites (**B**) detected in intra- and extracellular MΦ metabolite extracts (mean ± SD). Unpaired parametric t-tests with Welch’s correction (two-tailed; *p < 0.05; **p < 0.01; ***p < 0.001; ****p < 0.0001). Abbreviations denote: ADP, adenosine diphosphate; ATP, adenosine triphosphate; CDP, cytidine diphosphate; *CEPT1*, choline/ethanolamine phosphotransferase 1; *CHKA/B*, choline kinase α/β; CMP, cytidine monophosphate; CTP, cytidine triphosphate; DAG, diacylglycerol; *ETNK1/2*, ethanolamine kinase 1/2; *PHOSPHO1*, phosphoethanolamine/phosphocholine phosphatase; *PCYT1A/B*, phosphate cytidylyltransferase 1α/β; *PCYT2*, phosphate cytidylyltransferase 2.

Unexpectedly, M1 MΦs exhibited distinct metabolic changes associated with the choline and ethanolamine branches of the Kennedy pathway (Figure 5A), when compared to M0 and M2a MΦs. The profiling of MΦ intracellular metabolite extracts revealed a significant accumulation of choline and phosphoethanolamine in M1 MΦs (Figure 5B), where choline levels were increased 1.63 and 1.91-fold, and phosphoethanolamine levels were increased 2.00 and 2.03-fold relative to M0 and M2a MΦs, respectively (Table 1 and Table S2). Furthermore, intracellular phosphocholine levels were significantly decreased, –9.23 and –9.51-fold, in M1 MΦs relative to M0 and M2a MΦs, respectively (Figure 5B, Table 1, and Table S2). The profiling of MΦ extracellular metabolite extracts revealed similar trends with respect to choline levels (Figure 5B), with M0, M1, and M2a MΦ spent media containing an average surplus of 13.83, 52.20, and 6.41 nmol choline/mg protein, respectively (Table 2), relative to sham extracellular metabolite extract controls. Conversely, extracellular phosphocholine levels were significantly higher in M1 and M2a MΦs relative to M0 MΦs, and M2a MΦs had significantly higher levels of extracellular phosphocholine compared to M1 MΦs (Figure 5B); this amounted to average deficits of –81.15, –50.46, and –42.20 nmol phosphocholine/mg protein in the spent media of M0, M1, and M2a MΦs, respectively (Table 2), compared to sham extracellular metabolite extract controls. These data suggest that M1 MΦs repurpose the Kennedy pathway by diverting phosphocholine to choline, secreting excess choline into the media, and allowing for the accumulation of intracellular phosphoethanolamine.

## 3. Discussion

Several studies have highlighted the metabolic differences between M1 and M2 MΦs. While M1 MΦs appear to rely substantially on glycolysis for ATP production, M2 MΦs display greater dependence on mitochondrial ATP biogenesis and oxidative phosphorylation [9,17,18,28,29]. In addition, fatty acid synthesis predominates in M1 MΦs, whereas fatty acid beta oxidation seems to be preferentially associated with M2 MΦ phenotypes [17,22,30]. Thus, these studies explicitly suggest that the modulation of metabolism is vital to MΦ activation. However, most previous studies employed *in vitro* cell culture models using immortalized murine and human cell lines, and bone marrow-derived murine MΦs [17]. Comparable metabolomics studies of physiologically relevant human MΦ populations, such as primary human monocyte-derived MΦs, upon activation have been lacking. Although donor-dependent variation has been reported for human peripheral-blood cells, this donor heterogeneity has been demonstrated to be both stimulus- and cell subset-specific, with monocytes and B cells presenting lower inter-donor immune response variation compared to cytotoxic and helper T cells [31,32,33]. Our results from this study do not indicate any significant inter-donor variation; however, we have observed substantial inter-donor variability in primary human neutrophil metabolic adaptations upon stimulation, the data from which is currently being analyzed and assembled into another manuscript to be considered for publication, and have developed and implemented control and normalization methods to mitigate this issue. In the present study, we found that characteristic metabolic profiles clearly differentiate between human monocyte-derived M0, M1, and M2a MΦs. Diagnostic pathways include significant perturbation of glycolysis, lactate fermentation, the TCA cycle, oxidative stress, and *de novo* glycerophospholipid synthesis, which is also referred to as the Kennedy pathway [34].

The upregulation of glycolysis (Figure 2A) is viewed as a metabolic hallmark of M1 MΦ activation, which is critical for the modulation of M1 MΦ effector functions, including phagocytosis, pro-inflammatory cytokine secretion, and ROS production [18,19,20]. In our study, we established that M1 MΦs consume significantly greater amounts of carbohydrate substrates, such as fructose, glucose, and mannose, while concurrently secreting significantly greater amounts of lactate into the cell culture medium relative to M0 MΦs. These findings are consistent with previously reported results, and provide further evidence that pro-inflammatory MΦ activation induces Warburg-like metabolic traits [9,18,28]. Notably, we discovered that M2a MΦs exhibit similar glycolytic trends relative to M0 MΦs, albeit to a lesser extent than M1 MΦs. Although predominantly demonstrated in inflammatory M1 MΦs, our study demonstrates that glycolysis is also significantly upregulated in anti-inflammatory M2a MΦs. Previous studies had reported that M-CSF enhances the expression of genes encoding glucose transporters and glycolytic enzymes, in addition to promoting greater lactate production in M2 MΦs [35,36]. The addition of M-CSF in our *in vitro* differentiation model of primary human monocyte-derived MΦs may account for the differences observed between our study and other published reports with respect to M2 MΦ metabolism [28,37]. Our metabolomics results also indicate that increased glycolytic activity and lactate production may be universal markers of MΦ activation in primary human monocyte-derived M1 and M2a MΦs.

In addition to supporting the generation of the glycolytic intermediate fructose 6-phosphate (F6P), mannose can be used for the synthesis of N-glycans (Figure S3), which are important elements of protein glycosylation. Although N-glycosylation is well-established as a critical upregulated pathway in M2 MΦs [38], other studies have demonstrated that the inhibition of N-glycosylation also leads to the slight inhibition of M1 MΦ polarization [9]. Since our M1 and M2a MΦs both displayed a significant consumption of extracellular mannose relative to M0 MΦs, we examined these trends in the context of N-glycan biosynthesis, using microarray transcriptomics information that has been reported in Zhang et al. (Figure S3, Table S3) [39]. Bone marrow-derived murine MΦs stimulated with IFN-γ or LPS exhibit reduced gene expression of phosphomannomutase 1 (*Pmm1*), whereas IL-4-stimulated murine MΦs exhibit elevated gene expression of *Pmm1* relative to unstimulated control MΦs (Figure S3, Table S3) [39]. No consistent trend concerning guanosine diphosphate (GDP)-mannose pyrophosphorylase A (*Gmppa*) gene expression was reported in the Zhang et al. microarray data. However, IFN-γ, IL-4, and LPS-stimulated murine MΦs all exhibited elevated transcript expression of *Pmm2* and *Gmppb*, with the exception of the 2 and 4-hr time points following IL-4 stimulation, relative to unstimulated control MΦs (Figure S3, Table S3) [39]. These published transcriptomics data, combined with our metabolic observations regarding extracellular mannose consumption, thus suggest that M1 and M2a MΦs may utilize mannose for N-glycan biosynthesis and protein glycosylation, in addition to being used to provide substrates for glycolysis.

The utilization of a fully functional TCA cycle (Figure 3A) and concomitant oxidative phosphorylation is recognized as a metabolic characteristic of M0 and M2 MΦs [8,9,17], whereas M1 MΦs have been shown to reprogram this pathway to permit the accumulation of TCA cycle intermediates, including citrate and succinate, which are crucial for M1 MΦ effector functions [17,40]. Although previous studies have focused on TCA cycle intermediate-associated metabolic differences between M1 and M2a MΦs [40,41], investigations with respect to additional carbon sources that can enter the TCA cycle at the acetyl-CoA entry point are lacking. The metabolic data generated in this study suggest that M1 and M2a MΦs display distinct catabolic trends with respect to TCA cycle utilization. Both M1 and M2a MΦs exhibited preferential consumption of extracellular 3-hydroxybutyrate and acetate over pyruvate, relative to M0 MΦs. However, M1 MΦs utilized 3-hydroxybutyrate to a greater extent than M2a MΦs, and M2a MΦs utilized acetate to a greater extent than M1 MΦs. In addition, M1 and M2a MΦs secreted significant amounts of alanine and glycine into their spent culture medium, relative to M0 MΦs. These findings suggest that while M0 MΦs favor pyruvate as a carbon source for ATP energy production, M1 and M2a MΦs appear to prefer 3-hydroxybutyrate and acetate, respectively. This may be due, in part, to the fact that acetyl-CoA derived from 3-hydroxybutyrate coincidentally generates succinate, which is a known metabolic marker of M1 MΦs. Furthermore, acetyl-CoA derived from acetate utilizes ATP and not NAD^+^, the latter being needed to keep glycolysis going, an observation which is also supported by our glycolytic metabolic data for M1 and M2a MΦs (Figure 2). Previous studies have shown that bone marrow murine-derived MΦs stimulated with LPS exhibit elevated transcript expression of genes coding for 3-hydroxybutyrate dehydrogenase 1 (*Bdh1*) and 3-oxoacid-Co-A transferase 2 (*Oxct2*) enzymes at 24 hrs post-stimulation (Figure S4, Table S3) [39]. Although our intracellular succinate data for M1 MΦs do not reflect that expected from increased OXCT activity, it is important to note that our MΦs were stimulated for 72 hrs; therefore, without conducting additional temporal MΦ activation studies at time points shorter than 72 hrs, it is difficult to make any firm conclusions regarding differential 3-hydroxybutyrate consumption in our MΦ activation subtypes. The secretion of alanine and glycine, which takes place to a greater extent in M1 than M2a MΦs, may be a reflection of enhanced GPT and AGXT activity, with the concurrent generation of α-KG (Figure 3A), which is an important TCA cycle intermediate that has also been implicated in MΦ activation [17,40,42]. Altogether, these results suggest that M0, M1, and M2a MΦs preferentially utilize different carbon and amino acid sources that connect to the TCA cycle.

Additional enzymes that are critical for the flow of intermediates through the TCA cycle include pyruvate carboxylase (PC) and the malic enzyme (ME), which catalyze the conversion of pyruvate to oxaloacetate and malate to pyruvate, respectively (Figure 3A). The upregulation of these metabolic enzymes can occur when the flow of metabolites through the enzymatic reaction of pyruvate dehydrogenase (PDH; Figure 3A) is diminished, and when lactate fermentation is employed as a primary means to generate energy (ATP), a process referred to as the Warburg effect and which has been demonstrated in previous studies to take place in proliferating cancer cells (i.e., occurrence of aerobic glycolysis) [43,44,45]. Furthermore, increased activity of PC under glutamine-limited conditions has been previously shown to take place in glioblastoma and T cells [46,47]. Although our M1 and M2a MΦs display characteristics of Warburg metabolism (Figure 2), previous work by Meiser et al. has shown that PDH activity is unaffected in M1 MΦs [41]. Although our studies did not detect oxaloacetate or malate in our MΦ intracellular metabolite profiles, extracellular glutamine in our M0, M1, and M2a MΦ cultures was not found to be limiting upon MΦ harvest, remaining at extracellular concentrations of 0.92 ± 0.12, 1.37 ± 0.08, and 0.94 ± 0.05 mM, respectively, in the three MΦ subtypes. Our data leads us to conclude that replenishment of TCA cycle intermediates via the pyruvate carboxylase or malic enzyme reactions does not take place to a significant degree under the experimental conditions tested in our study.

Succinate is a key metabolic marker of M1 MΦ activation, and previous studies have established that its accumulation leads to the induction of Warburg-like metabolism in MΦs due to the stabilization of HIF-1α, which has also been shown to promote the upregulation of pro-inflammatory cytokine and gene expression [5,6]. Our M1 MΦ metabolic data are inconsistent with that shown by others in that we observed no significant difference between intracellular succinate levels in M1 MΦs relative to M0 MΦs; however, M2a MΦs do exhibit significantly decreased levels of intracellular succinate compared to M1 MΦs. Furthermore, we did not detect itaconate, which has been demonstrated to be an inflammatory regulator in murine and human MΦs [6,48], in any of our intra- or extracellular MΦ activation state metabolite extracts. These discrepancies may be due to the fact that our MΦs were activated for 72 hrs prior to metabolite extraction, while other studies typically employ shorter incubation periods, such as less than or up to 24 hrs. Notably, we also found that M1 MΦs display significantly decreased intracellular levels of fumarate. This suggests that our M1 MΦs may experience some level of succinate dehydrogenase (SDH) inhibition, as previously determined by others [5,40,49], since M1 MΦs exhibit a higher intracellular succinate to fumarate ratio than either M0 or M2a MΦs. Thus, these data would indicate that M1 MΦs attenuate their inflammatory response following 72 hrs of stimulation with LPS and IFN-γ. However, our results are consistent and in agreement with our other findings, as discussed below.

In addition to being derived from glutamate as a result of AGXT enzyme activity, glycine can also be produced from choline, serine, or alanine conversion (Figure S5). Unfortunately, the only available transcript data from Zhang et al. relevant to these metabolic pathways is that of *Agxt2*, which can catalyze the conversion of both alanine and glutamate to glycine [39]. Examining the microarray data, we found that *Agxt2* gene transcription is upregulated at 24 hrs in IFN-γ and LPS-stimulated murine MΦs, and is downregulated at 24 hrs in IL-4-stimulated murine MΦs, relative to unstimulated control murine MΦs (Figure S5, Table S3) [39]. A further investigation of our relevant metabolic data revealed that M2a MΦs consume significantly less extracellular serine than M0 or M1 MΦs. In addition, M2a MΦs displayed significantly decreased levels of intracellular serine (Figure S5). Furthermore, M2a MΦs consumed significantly less extracellular glutamate than M0 or M1 MΦs (Figure S5). Overall, these metabolic findings, combined with the microarray data of Zhang et al., thus suggest that glycine is derived primarily from glutamate as a result of AGXT2 enzyme activity in M1 MΦs, while M2a MΦs may be producing glycine primarily from serine using serine hydroxymethyltransferase (SHMT), although further studies are needed to validate the interpretations of these data.

Extracellular secreted fumarate, at a concentration of 3.95 ± 0.73 µM, was only found in our M2a MΦ cultures (Figure 3B). These findings are consistent with studies that have shown that fumarate is a key metabolite for memory induction in innate immune cells, which is also known as trained immunity [50]. Arts et al. determined that the training of primary human monocyte-derived MΦs using 100 µM of fumarate induced changes >2.5-fold relative to non-trained MΦs, in 456 dynamic epigenetic regions as a result of histone modifications, such as H3K4me3 and H3K27ac [50]. Many of these dynamic fumarate-induced epigenetic alterations were found to be associated with cellular pathways involved in leukocyte migration and the innate immune response, including pro-inflammatory cytokine expression [50]. Findings by Arts et al. are also consistent with previous trained immunity studies, which used β-glucan-trained monocytes [51,52]. Additional studies have shown that fumarate treatment induces IL-4 production and T_H_2 responses in human type II dendritic cells and CD4^+^ T cells [53]. Therefore, these published data, together with our observation of significant levels of extracellular fumarate in our human M2a MΦ cell cultures, would suggest that our M2a MΦs may be undergoing local immunological memory and T_H_2-like cell responses.

Microbicidal ROS production is a well-characterized phenomenon of MΦ function and immune responses. Nevertheless, excessive ROS production can lead to impaired intracellular redox balance, a depletion of intracellular pools of GSH due to excessive conversion of GSH to its oxidized form (GSSG), NADPH depletion, lipid and protein peroxidation, and oxidative DNA damage (Figure 4A), which, if left unchecked, can lead to significant cellular damage and apoptosis [54,55]. Our study indicates that M1 and M2a MΦs experience significant oxidative stress, as revealed by significantly decreased levels of intracellular GSH relative to M0 MΦs. Furthermore, M1 MΦs have significantly lower intracellular levels of NADPH relative to M0 MΦs. These results are consistent with other published works, which have reported that NADPH oxidase (NOX) produces superoxide (^•^O_2_^-^) from molecular oxygen (O_2_) using NADPH produced during the oxidative phase of the pentose phosphate pathway (PPP), which is upregulated in M1 MΦs [9,17,54]. In addition, NADPH provides a source of reducing power to regenerate GSH from GSSG, thereby indirectly contributing to ROS neutralization [17,56]. Such a process seems to account for the significantly decreased intracellular levels of GSH that we have observed in M1 MΦs relative to M2a MΦs. Although ROS production is associated predominantly with M1 MΦ phenotypes, another published report demonstrated that Cu,Zn-superoxide dismutase (SOD)-mediated hydrogen peroxide (H_2_O_2_) production supports M2 MΦ activation via redox-dependent STAT6 nuclear translocation, which results in reduced TNF-α expression and elevated profibrotic factor gene expression [57]. These reports are consistent with our metabolomics findings, which indicate that primary human monocyte-derived M2a MΦs experience an oxidative stress response, which may be due to low-level ROS production in these MΦs. We also found that M1 MΦs contain a significantly greater intracellular amount of taurine compared to M0 and M2a MΦs. Similar to reduced glutathione (GSH), taurine exhibits cytoprotective effects by neutralizing toxic oxidative species and attenuating excessive oxidative stress responses [55,58]. Collectively, our findings indicate that MΦ activation results in significant oxidative stress, and suggest that M1 MΦs deplete intracellular NADPH for ROS production and GSH regeneration, in addition to accumulating intracellular taurine to mitigate ROS-mediated M1 MΦ cell damage.

Previous studies have shown that M1 and M2 MΦs have disparate metabolic preferences concerning lipid metabolism, with fatty acid synthesis being associated with M1 MΦ activation and fatty acid beta oxidation being preferentially associated with M2 MΦ activation [1,7,17,22]. In this study, we found that M1 MΦs display unique metabolic markers related to *de novo* glycerophospholipid synthesis, which is also known as the Kennedy pathway (Figure 5A). M1 MΦs exhibited significantly increased levels of intra- and extracellular choline and significantly decreased levels of intracellular phosphocholine, relative to M0 and M2a MΦs. Snider et al. showed that M1 MΦs consume greater amounts of choline, promote phosphatidylcholine biosynthesis, and that an antibody-mediated inhibition of choline uptake altered M1 MΦ secretion of pro-inflammatory cytokines [59]. While our results differ from this published work, our M1 MΦs were stimulated for 72 hrs prior to metabolic analysis, whereas Snider et al. used much shorter LPS incubation times, ranging from 10 min to 16 hrs [59], which may account for the different observations. In addition, other studies have determined that choline reduces inflammation, IL-1β release from innate immune cells, pro-inflammatory gene expression, such as *TLR4*, *NFKB1*, and *TNFA*; and increases lymphocyte proliferation [60,61,62]. Thus, our M1 MΦ data suggest that at 72 hrs post-activation, these cells display metabolic trends that are associated with inflammatory mitigation. We also found that M1 MΦs accumulate significant amounts of intracellular phosphoethanolamine, which was not observed in M0 or M2a MΦs. Gohil et al. demonstrated that mammalian cells elevate intracellular phosphoethanolamine levels following meclizine treatment due to the dose-dependent, non-competitive inhibition of phosphate cytidylyltransferase 2 (PCYT2) enzymatic activity [63]. Furthermore, it was also determined that phosphoethanolamine reduces mitochondria membrane potential, increases flavin adenine dinucleotide (FAD^+^), and decreases reduced nicotinamide adenine dinucleotide (NADH) levels, as a result of mitochondrial respiration inhibition [63]. Examining supplementary microarray transcriptomics data included in Zhang et al., we discovered that bone marrow-derived murine MΦs stimulated with IFN-γ or LPS have a reduced expression of *Chkα*, *Chkβ*, *Pcyt1a*, and *Pcyt2* transcripts and elevated expression of *Etnk1*, whereas bone marrow-derived murine MΦs stimulated with IL-4 exhibit a reduced expression of *Chkα* and *Chkβ* transcripts and elevated gene expression of *Etnk1*, *Pcyt1a*, and *Pcyt2* relative to unstimulated control MΦs (Figure 5A, Figure S6, and Table S3) [39]. These transcriptomics profiles are consistent with our metabolomics findings, and together suggest that M1 MΦs distinctly manipulate the Kennedy pathway to downregulate mitochondrial respiration and to attenuate their inflammatory response, at least at the 72-hr stimulation time point used in this study.

Quinolinate, an N-methyl-D-aspartate (NMDA) receptor agonist and neurotoxin, is a metabolite derived from tryptophan within the kynurenine pathway (KP) [64]. Our results indicate that intracellular metabolite extracts from M1 MΦs contain significantly greater amounts, 25.90-fold compared to M0 MΦs, of quinolinate (Table 1). This is consistent with previous studies that have demonstrated substantial quinolinate production by macrophages following IFN-γ stimulation [64,65]. At concentrations less than 50 nM, quinolinate serves as a substrate for NAD^+^ synthesis by the KP; however, at concentrations above 150 nM, quinolinate can induce excitotoxicity in astrocytes and neurons, induce NOS, and cause oxidative stress [66]. Quinolinate is able to form complexes with Fe(II), which promotes the formation of ROS, such as hydroxyl (^•^OH) radicals, and subsequent DNA degradation and lipid peroxidation [67]. More recent evidence has emerged indicating that KP activation is triggered upon innate immune challenge in MΦs; yet, the conversion of quinolinate to NAD^+^ is oddly inhibited [68]. This particular study also found that oxidative phosphorylation in activated MΦs can be restored by increasing *de novo* NAD^+^ synthesis by the KP, which implies that this process is a metabolic switch for MΦ effector function [68]. Our M1 MΦs also displayed significantly depleted levels, –3.79-fold compared to M0 MΦs, of intracellular NAD^+^; therefore, we believe that these findings suggest that M1 MΦs may accumulate intracellular quinolinate as another means to inhibit oxidative phosphorylation and/or generate ROS.

While O_2_ consumption was not directly measured in our MΦ subtype NMR metabolomics experiments, the metabolome profiles of M0, M1, and M2a primary MΦs that we have characterized in this study are consistent with published reports on this subject. An interesting future direction for this work would be to track the metabolome changes of our different MΦ subtypes as a function of time, i.e., 24, 36, and 48 hrs, rather than at the single 72 hrs time point that was employed in this current study. We would expect to detect interesting metabolic adaptations as the MΦs activate over time in this *in vitro* cell culture model. In the future, we aim to conduct these O_2_ consumption measurements in parallel with longitudinal metabolomics analyses, as well as to report on the metabolic adaptations of MΦs when co-cultured with bacterial pathogens.

## 4. Materials and Methods

### 4.1. Primary Human Monocyte Isolation

Heparinized whole blood was obtained in accordance with proper guidelines, local Institutional Review Board (IRB) approval (ID# 00000799; Protocol #VC100118), and informed consent from healthy, adult donors in Bozeman, Montana, USA. A total of 6 donors, in the age range of 19 to 27 were included in this study, 50% of whom were female. Peripheral blood mononuclear cells (PBMCs) were isolated by centrifugation in lymphocyte separation media (Corning) at 800× *g* for 25 min at room temperature. CD14^+^ monocytes were isolated from PBMCs by MACS using CD14 human microbeads (Miltenyi Biotec, San Diego, CA, USA, which resulted in an average purity of 97.1 ± 1.3% (Figure S7) when subjected to sorting on an LSR Fortessa cell analyzer (BD Biosciences San Jose, CA, USA).

### 4.2. Culture of Primary Human Monocyte-Derived MΦs

To generate primary human monocyte-derived MΦs, CD14^+^ monocytes were cultured in 25 cm^2^ tissue culture flasks (1 × 10^6^ cells per mL; Falcon/Corning) in Roswell Park Memorial Institute (RPMI) medium -1640, w/L-glutamine (Lonza, Bend, OR, USA) media, supplemented with 1 mM of sodium pyruvate (Lonza), 1X MEM non-essential amino acids (NEAAs; Gibco, ThermoFisher Scientific, USA), 10% (v/v) fetal bovine serum (FBS; ATCC, Manassas, VA, USA), and 50 ng/mL recombinant human M-CSF (rHU-MCSF; PeproTech, Rocky Hill, NJ, USA) for 6 d. Media and cytokines were replenished every 3 d.

### 4.3. Activation of Primary Human Monocyte-Derived MΦs

Mature (6 d) monocyte-derived MΦs were prepared in 3 replicates using 25 cm^2^ tissue culture flasks (Falcon/Corning) containing RPMI 1640, w/L-glutamine (Lonza) media, supplemented with 1 mM of sodium pyruvate (Lonza), 1X MEM NEAAs (Gibco), 10% (v/v) FBS (ATCC), and 50 ng/mL rHU-MCSF (PeproTech). Three different MΦ activation groups were assessed and compared, including M0, M1, and M2a MΦs. Cell cultures corresponding to each MΦ activation state, and parallel sham media controls, corresponding to macrophage activation and cell culture media added to 25 cm^2^ tissue culture flasks that did not contain mature monocyte-derived MΦs, were set up and incubated for 72 hrs at 37 °C, 5% CO_2_. MΦ activation stimuli included 100 ng/mL of LPS and 50 ng/mL of recombinant human IFN-γ (rHU-IFNγ) for M1 MΦs (Sigma Aldrich and PeproTech, respectively), whereas no additional stimuli were added to M0 MΦs. Then, 20 ng/mL recombinant human IL-4 (rHU-IL4; PeproTech) was utilized to generate M2a MΦs. Following culture and activation, the phenotypic characterization of primary human monocyte-derived MΦs (MoMΦs) was conducted using flow cytometry (Figure S8).

### 4.4. Antibodies and Flow Cytometry

Primary human monocytes were stained using anti-human antibodies directed against CD14 (BD Biosciences) and appropriate isotype controls. M0, M1, and M2a MoMΦs were stained using anti-human antibodies directed against CD68, CD80, and CD163 (BD Biosciences) and appropriate isotype controls. Permeabilization of a cell subset was conducted using Cytofix/Cytoperm (BD Biosciences) for the intracellular staining of CD68. Following staining, cells were resuspended in fluorescence-activated cell sorting (FACS) buffer for analysis on an LSR Fortessa Flow Cytometer (BD Biosciences). FACS data were analyzed using FCSalyzer software (version 0.9.14-alpha) with monocytes and MoMΦs gated based upon size and single cells. Since baseline fluorescence levels differed between MΦ activation states, mean fluorescence intensity (MFI) values were normalized by subtracting the appropriate isotype control MFI from the sample MFI (normalized MFI; Figure S8).

### 4.5. Intra- and Extracellular Metabolite Extraction

Following MΦ activation, spent cell culture medium was transferred from 25 cm^2^ tissue culture flasks into sterile 15-mL conical tubes and centrifuged at 2000× *g* for 1 min at room temperature (RT) to pellet any cellular debris. Two 1.5-mL aliquots of sham and spent cell culture medium were transferred to sterile 1.5-mL tubes and stored at –80 °C prior to extracellular metabolite extraction. Cell monolayers were washed with 1 mL of cold (4 °C) sterile 1X phosphate-buffered saline (PBS). Wash solutions were pipetted into a 15-mL conical tube, centrifuged at 2000× *g* to pellet any remaining non-adherent cells, decanted, and then resuspended in 500 µL of –20 °C 50% aqueous methanol. Then, 1.5 mL of –20 °C 50% aqueous methanol was added to 25 cm^2^ tissue culture flasks to simultaneously quench, extract, and detach cells from the flask surface with the aid of a cell scraper. Cell suspensions were removed from 25 cm^2^ tissue culture flasks, transferred to 15-mL conical tubes containing non-adherent cell suspensions, and thoroughly mixed.

Two aliquots of 1-mL cell suspensions were transferred to two separate 2-mL lysis B matrix tubes (MP Biomedicals) and lysed using a FastPrep-24 5^G^ homogenizer (MP Biomedicals) at a speed of 6.0 m/s for 2 cycles of 40 s each, with lysis tubes placed on ice between cycles. Then, 50 µL of cell lysate was stored at –80 °C for protein determination, and then 500 µL of chloroform (CHCl_3_) was added to each lysis tube. Tubes were vortexed for 3 cycles of 10 s each, and then placed at –20 °C for 20 min, prior to centrifugation at 10,000× *g* for 10 min to separate aqueous and nonpolar phases [69]. The aqueous phase, containing the intracellular metabolite mixture, was transferred to a 1.5-mL tube, dried using a Speedvac vacuum centrifuge with no heat overnight, and stored at –80 °C until NMR metabolite sample preparation.

Sham and spent cell culture medium samples were filtered through 3-kDa molecular weight cutoff (MWCO) centrifuge filters (Millipore Amicon), which were prewashed extensively [70], prior to being dried using a Speedvac vacuum centrifuge with no heat overnight and stored at –80 °C until NMR sample preparation.

### 4.6. Determination of Protein Content

Protein concentrations were measured using the Pierce BCA Protein Assay Kit (ThermoFisher Scientific; Cat. No. 23225) for the normalization of intra- and extracellular metabolite extracts.

### 4.7. NMR Sample Preparation

Dried intra- and extracellular metabolite extracts were resuspended in 600 µL of NMR buffer [consisting of 25 mM of NaH_2_PO_4_/Na_2_HPO_4_, 0.4 mM of imidazole, 0.25 mM of 4,4-dimethyl-4-silapentane-1-sulfonic acid (DSS) in 90% H_2_O/10% D_2_O, pH 7.0]. Following resuspension, samples were centrifuged at 21,000 rpm for 1 min to pellet insoluble debris, and then transferred to 5-mm NMR tubes for NMR metabolomics analysis.

### 4.8. NMR Spectra Acquisition and Preprocessing

All NMR spectra were collected at 298 K (25 °C) using a Bruker 600 MHz (^1^H Larmor frequency) AVANCE III solution NMR spectrometer, equipped with a SampleJet automatic sample loading system, a 5-mm triple resonance (^1^H, ^15^N, ^13^C), liquid-helium-cooled three-channel inverse (TCI) detection NMR cryoprobe, and Topspin software (Bruker version 3.2). Then, 1D ^1^H NMR spectra acquisition was performed using the Bruker-supplied excitation sculpting (ES)-based ‘zgesgp’ pulse sequence [71,72], and NMR spectra were recorded with 256 scans and a ^1^H spectral window of 9615.38 Hz. Free induction decays (FIDs) were collected with 32K data points and a dwell time interval of 52 µsec, amounting to a data acquisition time of 1.7 s. Recovery delay (D1) times between acquisitions were set to 1 s, resulting in an overall 2.7 s relaxation recovery delay between scans [73,74]. DSS chemical shift referencing and phase correction of 1D ^1^H NMR spectra were conducted using Topspin software (Bruker version 3.2).

For the verification of select metabolite identifications, 2D ^1^H–^1^H total correlation spectroscopy (TOCSY) spectra were acquired for representative samples using the Bruker-supplied ‘mlevphpr.2/mlevgpph19′ pulse sequences (256 × 2048 data points, 2 s relaxation delay, 32 transients per FID, ^1^H spectral window of 6602.11 Hz, 80 ms TOCSY spin lock mixing period). Then, 2D ^1^H-^1^H TOCSY spectra were processed using Topspin software (Bruker version 3.2).

### 4.9. NMR Data Analysis

Further processing of 1D ^1^H NMR spectra and metabolite profiling analyses were conducted using Chenomx NMR Suite software (version 8.1; Chenomx Inc., Edmonton, Alberta, Canada). The baseline correction of NMR spectra following an import of preprocessed ‘1r’ NMR spectral files into Chenomx software was performed using the automatic cubic spline function in Chenomx, and subsequent manual breakpoint adjustment to obtain a flat, well-defined baseline, following recommendations from Chenomx application notes and previously reported methods [75,76]. ^1^H chemical shifts were referenced to the 0.0 ppm DSS signal, and the ^1^H NMR signals arising from imidazole were used to correct for small chemical shift changes due to slight variations in sample pH. Metabolite identification and quantitation were performed by fitting the 1D ^1^H spectral patterns, chemical shifts, and spectral intensities to reference spectral patterns of small molecules, using the Chenomx small molecule spectral database for 600 MHz (^1^H Larmor frequency) magnetic field strength NMR, and a manual peak-based fit style, where adjustments were made to achieve optimal fits for compound peak cluster location and intensity [77]. An internal (0.25 mM DSS) standard was used for metabolite quantitation.

Although pulse programs utilizing ES suppress proton signals around the water region to a greater extent than NOEPR (1D NOESY with presaturation during relaxation and mixing time), the relative intensities observed between these particular ^1^H signals have been demonstrated to be identical to those seen in NOEPR [78]. To evaluate the differences between the 1D ^1^H spectra acquired using ‘zgesgp’ versus ‘noesypr1d’ pulse sequences, we have conducted comparative analyses by constructing our own in-house ‘zgesgp’-acquired 600 MHz metabolite library using pure standards. If our in-house metabolite standard 1D ^1^H NMR spectra presented significant deviations from the 600 MHz Chenomx small molecule spectral database, we added these ‘zgesgp’ metabolite standard spectra to a custom library using the ‘Compound Builder’ module of Chenomx NMR Suite program (version 8.0), as described previously [74].

### 4.10. Statistical Analysis

Quantitative intra- and extracellular metabolic profiles were exported from Chenomx software, as mM concentrations, with metabolic profiles generated in parallel from blank NMR buffer control samples subtracted from our experimental MΦ extract profiles. In addition, metabolite profiles generated from parallel sham media controls were subtracted from our extracellular MΦ extract metabolite profiles. Intra- and extracellular metabolite concentrations were converted from mM to nmol by accounting for NMR buffer volume, and resulting metabolic profiles were normalized to protein content prior to multivariate statistical analysis using MetaboAnalyst 4.0 [79,80]. Normalized metabolite concentrations were further log-transformed to ensure a Gaussian distribution of the data and auto-scaled (i.e., mean centered and divided by standard deviation) prior to statistical analysis, including 2D-PCA and HCA. The false discovery rate (FDR) was evaluated using the ‘ANOVA’ module of MetaboAnalyst, an adjusted *p*-value (FDR) cutoff of 0.05, and Tukey’s HSD post-hoc analysis. Metabolites with FDR values ≥0.05 were excluded from further statistical analysis in GraphPad Prism. HCA was conducted in MetaboAnalyst using a Euclidean distance measure and Ward clustering algorithm. Statistical significance was assessed by an unpaired parametric *t*-test with Welch’s correction using the GraphPad Prism program version 8.0.2 (GraphPad Software, La Jolla, CA).

## 5. Conclusions

In conclusion, our metabolite profiling data generated using *in vitro* MΦs derived from primary human monocytes indicate that M1 and M2a MΦs both utilize glycolysis and exhibit significant oxidative stress. Furthermore, the M1 MΦs generated in this study reveal a previously unknown and unique repurposing of the *de novo* glycerophospholipid metabolic pathway associated with the inhibition of oxidative phosphorylation and inflammatory mitigation. Moreover, we identified unique metabolite expression patterns relevant to metabolite flow through the TCA cycle, including the preferential utilization of 3-hydroxybutyrate and acetate in M1 and M2a MΦs, respectively, rather than pyruvate. The results from our study emphasize the importance of investigating the biochemical properties of physiologically relevant innate immune cell populations, such as primary human monocyte-derived MΦs. Our work also highlights the usefulness of NMR metabolomics to define characteristic metabolic phenotypes (i.e., metabotypes) associated with specific cellular phenotypes, and to better understand how functionally relevant metabolic adaptations correspond to distinct, physiologically relevant activation states of primary human immune cells cultured *in vitro*.

## Figures and Tables

**Table 1:** Discriminatory metabolites in intracellular extracts associated with activation.

Metabolite	M1 MΦs		M2a MΦs	
	FC	*p*-Value	FC	*p*-Value
2-Oxoisocaproate	–1.28	NS	–1.88	**
Acetate	–1.66	*	–2.11	**
ADP	1.05	NS	1.42	***
AMP	1.33	**	2.43	****
Arginine	7.54	****	7.44	****
Aspartate	–1.60	**	–1.21	NS
ATP	9.14	****	ND	N/A
β-Alanine	3.01	****	2.53	***
Betaine	6.38	****	2.95	****
Choline	1.63	***	–1.18	NS
Creatine	–1.24	**	1.18	NS
Creatine phosphate	2.81	****	2.13	***
Fumarate	–1.68	***	1.11	NS
Glucose	–9.85	***	–6.68	**
Glucose-1 phosphate	–5.10	****	–5.10	****
Glutamate	–2.67	***	–1.22	NS
Glutamine	1.02	NS	–1.33	*
Glutathione	–1.81	**	–1.34	*
Glycerol	–3.10	***	–2.53	***
Glycine	1.27	**	1.21	*
GTP	–1.01	NS	1.48	***
Lactate	1.79	****	1.66	****
Lysine	–1.11	NS	–1.25	*
Myo-Inositol	–2.43	****	1.88	***
NAD^+^	–3.79	****	1.07	NS
NADPH	–2.40	**	1.34	*
Niacinamide	5.19	****	ND	N/A
O-phosphocholine	–9.23	****	1.03	NS
O-phosphoethanolamine	2.00	***	–1.02	NS
Propionate	–1.13	NS	–1.57	*
Quinolinate	25.90	****	ND	N/A
Serine	–1.12	NS	–1.67	*
Succinate	–1.23	NS	–1.82	**
Taurine	1.23	*	–1.18	*
Tyrosine	–1.20	NS	–1.50	*
UMP	–1.67	***	–1.04	NS

Metabolites were selected based upon fold change (FC) of intracellular metabolite concentrations, and statistical significance between activated MΦ subsets (M1 and M2a MΦs; nmol/mg protein; calculated from metabolite spectral fitting using the Chenomx NMR Suite software and the standard Chenomx 600-MHz metabolite library; ND, not detected) relative to M0 MΦs. Fold changes were calculated relative to M0 MΦs, whereby increases are shown as positive values and decreases are shown as negative values. Statistical significance (*p*) was measured using two-tailed unpaired parametric *t*-tests with Welch’s correction, whereby *, *p* < 0.05; **, *p* < 0.01; ***, *p* < 0.001; ****, *p* < 0.0001. Abbreviations denote: NS, not statistically significant; N/A, not applicable; ADP, adenosine diphosphate; AMP, adenosine monophosphate; ATP, adenosine triphosphate; GTP, guanosine triphosphate; NAD^+^, nicotinamide adenine dinucleotide; NADPH, reduced nicotinamide adenine dinucleotide phosphate; UMP, uridine monophosphate. Fold change (FC) values for arginine, ATP, betaine, glucose-1 phosphate, NAD^+^, niacinamide, and quinolinate in M1 MΦs and arginine, betaine, and glucose-1 phosphate in M2a MΦs were calculated using limit of detection (LOD) values (see Table S1) determined from M0 MΦ intracellular metabolite extract 1D ^1^H NMR spectra.

**Table 2:** Discriminatory metabolites in extracellular extracts associated with activation.

Metabolite	Concentration (Mean ± SD)			*p*-Value		
	M0 MΦs	M1 MΦs	M2a MΦs	M1 vs. M0	M2a vs. M0	M1 vs. M2a
2-Hydroxybutyrate	48.65 ± 13.52	132.89 ± 17.24	23.43 ± 7.29	****	**	****
2-Oxoisocaproate	60.64 ± 10.85	26.96 ± 5.31	2.81 ± 2.92	***	****	****
3-Hydroxybutyrate	–20.75 ± 13.78	–116.64 ± 8.81	–96.50 ± 3.85	****	****	**
3-Hydroxyisobutyrate	18.01 ± 3.36	16.87 ± 2.38	12.49 ± 2.03	NS	**	**
3-Methyl-2-oxovalerate	108.34 ± 49.08	61.13 ± 9.68	21.34 ± 3.60	NS	**	****
Acetate	39.73 ± 73.93	–150.67 ± 13.89	–213.14 ± 27.60	**	***	**
Alanine	–60.54 ± 73.40	98.11 ± 67.21	20.73 ± 35.65	**	*	*
Arabinose	–107.36 ± 12.28	–109.08 ± 12.55	–75.83 ± 4.50	NS	***	***
Arginine	852.67 ± 399.21	1349.61 ± 290.20	788.70 ± 198.71	*	NS	**
Aspartate	–791.19 ± 90.54	–797.31 ± 93.74	–573.21 ± 22.03	NS	**	**
Carnitine	37.60 ± 39.79	–10.46 ± 1.09	–9.89 ± 0.39	*	*	NS
Choline	13.83 ± 12.57	52.20 ± 9.38	6.41 ± 14.85	***	NS	***
Creatine	1.50 ± 4.85	–5.73 ± 5.69	3.80 ± 3.65	*	NS	**
Cysteine	–147.54 ± 22.37	–103.71 ± 24.21	–125.00 ± 11.15	**	NS	NS
Cystine	183.44 ± 50.04	419.86 ± 62.88	468.59 ± 62.40	****	****	NS
Formate	140.60 ± 46.15	195.50 ± 61.22	228.89 ± 66.43	NS	*	NS
Fumarate	ND	ND	9.51 ± 1.67	N/A	****	****
Glucose	–21044.40 ± 1478.98	–42208.15 ± 4621.45	–30552.65 ± 1595.13	****	****	**
Glutamate	–674.75 ± 78.82	–595.54 ± 116.43	–363.96 ± 18.48	NS	***	**
Glutamine	1235.12 ± 211.97	1976.33 ± 320.59	731.62 ± 127.38	**	***	****
Glycine	179.01 ± 122.04	404.88 ± 106.51	501.91 ± 115.32	**	***	NS
Histidine	41.76 ± 24.39	98.44 ± 23.25	56.36 ± 17.82	**	NS	**
Isoleucine	–78.84 ± 63.44	–13.97 ± 166.32	60.25 ± 54.71	NS	**	NS
Lactate	29467.61 ± 2714.66	51046.16 ± 4806.45	38150.09 ± 2610.21	****	***	***
Leucine	–141.51 ± 88.78	28.65 ± 126.92	19.93 ± 35.22	*	**	NS
Lysine	25.26 ± 51.14	325.04 ± 43.60	209.60 ± 20.31	****	***	***
Mannose	9.84 ± 12.78	–54.62 ± 7.85	–43.79 ± 7.40	****	****	*
O-phosphocholine	–81.15 ± 20.78	–50.46 ± 7.86	–42.20 ± 2.26	*	**	*
Phenylalanine	2.21 ± 11.13	26.02 ± 18.03	–9.66 ± 9.85	*	NS	**
Proline	–51.72 ± 53.94	60.38 ± 54.56	–27.75 ± 21.10	**	NS	**
Pyroglutamate	121.33 ± 345.73	–564.30 ± 551.92	–386.27 ± 161.22	*	*	NS
Pyruvate	–642.79 ± 67.35	–489.93 ± 121.02	–336.50 ± 17.62	*	****	*
Serine	–179.15 ± 49.58	–45.09 ± 75.77	–284.36 ± 89.47	**	*	***
Urea	–1521.10 ± 205.87	–2142.83 ± 454.07	–1152.24 ± 159.89	*	**	**
Valine	–25.75 ± 27.43	89.95 ± 97.95	10.00 ± 50.05	*	NS	NS

Metabolites were selected based upon the statistical significance of extracellular metabolite concentrations between activated MΦ subsets (M1 and M2a MΦs; nmol/mg protein; calculated from metabolite spectral fitting using the Chenomx NMR Suite software and the standard Chenomx 600 MHz metabolite library; ND, not detected) relative to M0 MΦs. Metabolite concentrations were normalized to sham media controls, whereby increases are shown as positive values and decreases are shown as negative values. Statistical significance (*p*) was measured using two-tailed unpaired parametric *t*-tests with Welch’s correction, whereby *, *p* < 0.05; **, *p* < 0.01; ***, *p* < 0.001; ****, *p* < 0.0001; NS, not statistically significant; N/A, not applicable. Limit of detection (LOD) values for fumarate in M0 and M1 MΦ extracellular metabolite extract 1D ^1^H NMR spectra have been provided for reference (see Table S1).

## Supplementary Materials

The following are available online at https://www.mdpi.com/2218-1989/9/11/248/s1, Figure S1. 1D ^1^H NMR spectra acquired on MSU’s Bruker 600 MHz (^1^H Larmor frequency) NMR spectrometer on intra- (A) and extracellular (B) metabolite extracts from M0 MΦs; Figure S2. PCA loadings plots for intra- (A) and extracellular (B) MΦ metabolite extracts; Figure S3. Metabolism of mannose for N-glycan biosynthesis (A) and fold change expression of *Gmppa*, *Gmppb*, *Pmm1*, and *Pmm2* in bone marrow-derived murine MΦs upon differential stimuli (B); Figure S4. Fold change expression of *Bdh1* (mMR028403), *Bdh1** (mMC011803), *Oxct1*, and *Oxct2* in bone marrow-derived murine MΦs upon differential stimuli; Figure S5. Generation of glycine from (A) choline or (B) glutamate, serine, and alanine; Figure S6. Fold change expression of *Chkα*, *Chkβ*, *Etnk1*, *Pcyt1a*, and *Pcyt2* in bone marrow-derived murine MΦs upon differential stimuli; Figure S7. Purity of isolated monocytes; Figure S8. Phenotype of primary human monocyte-derived macrophages (MoMΦs); File S1. Intracellular PCA loadings; File S2. Extracellular PCA loadings; Table S1. 1D ^1^H NMR intra- and extracellular metabolite limit of detection (LOD) values; Table S2. Discriminatory metabolites in intracellular extracts associated with M1 vs. M2a MΦ activation; Table S3. Relative fold change expression of select genes in bone marrow-derived murine MΦs upon differential stimuli.
