# Supplementary material for: Quantitative 1H NMR Metabolomics Reveal Distinct Metabolic Adaptations in Human Macrophages Following Differential Activation
Source: Metabolites. 2019 Oct 24;9(11):248. doi: 10.3390/metabo9110248 (PMC6918149; doi:10.3390/metabo9110248)
Supplement: Supplementary file 1 [file metabolites-09-00248-s001.pdf]

Supplementary Material

Quantitative ^{1}H NMR Metabolomics Reveal Distinct Metabolic Adaptations in Human Macrophages Following Differential Activation

Amanda L. Fuchs *, Sage M. Schiller, Wyatt J. Keegan, Mary Cloud B. Ammons †, Brian Eilers, Brian Tripet and Valérie Copié *

Department of Chemistry and Biochemistry, Montana State University, Bozeman, MT 59717, USA; sageschiller2@gmail.com (S.M.S.); wjkeegan@msn.com (W.J.K.); marycloud.ammonsanderson@va.gov (M.C.B.A.); bjeilers@gmail.com (B.E.); brian.tripet@gmail.com (B.T.)

† Current Address: Boise Veterans Medical Center, Idaho Veterans Research and Education Foundation, Boise, ID, 83702 USA

* Correspondence: afuchs03143@gmail.com (A.L.F.); vcopie@montana.edu (V.C.); Tel.: 406-946-2073 (A.L.F.); 406-994-7244 (V.C.);

Received: 5 September 2019; Accepted: 22 October 2019; Published: 24 October 2019

Amanda L. Fuchs *et al.*, Figure S1

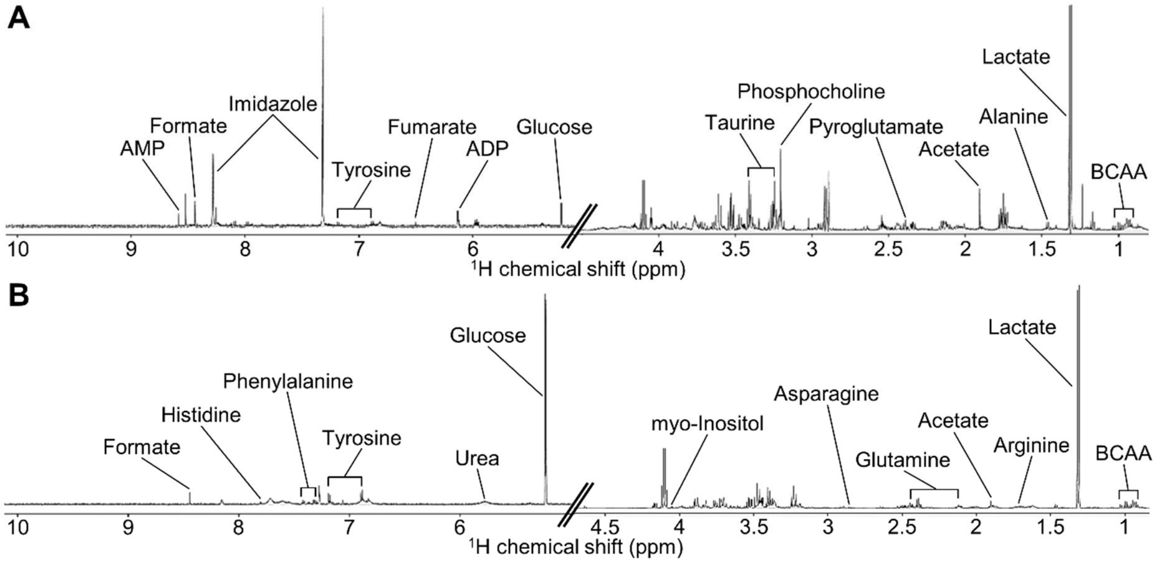

Figure S1. 1D ^{1}H NMR spectra acquired on MSU's Bruker 600 MHz (^{1}H Larmor frequency) NMR spectrometer on intra- **(A)** and extracellular **(B)** metabolite extracts from M0 MΦs. Abbreviations denote: AMP, adenosine monophosphate; ADP, adenosine diphosphate; BCAA, branched chain amino acids.

Amanda L. Fuchs *et al.*, Figure S2

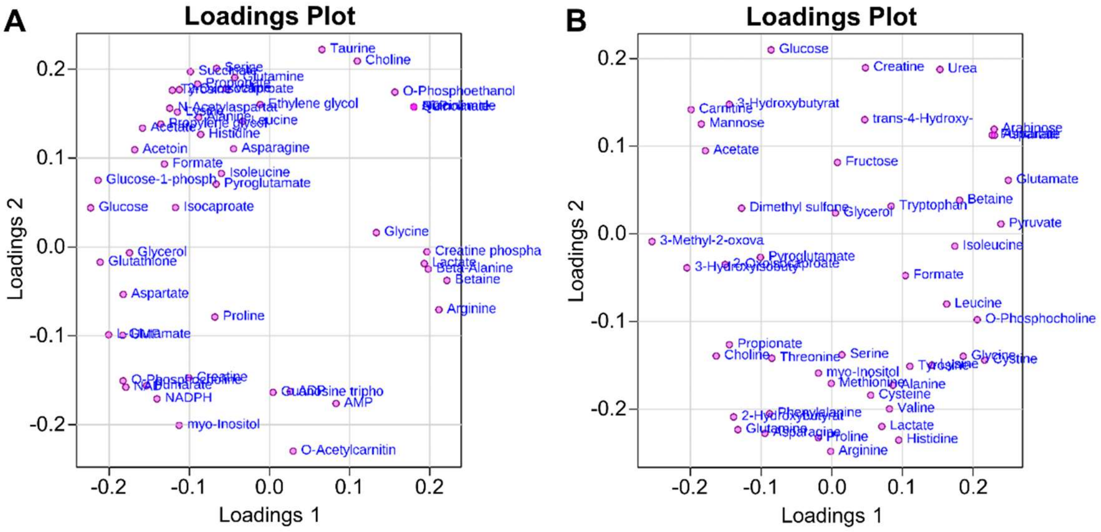

Figure S2. PCA loadings plots for intra- **(A)** and extracellular **(B)** MΦ metabolite extracts.

Amanda L. Fuchs *et al.*, Figure S3

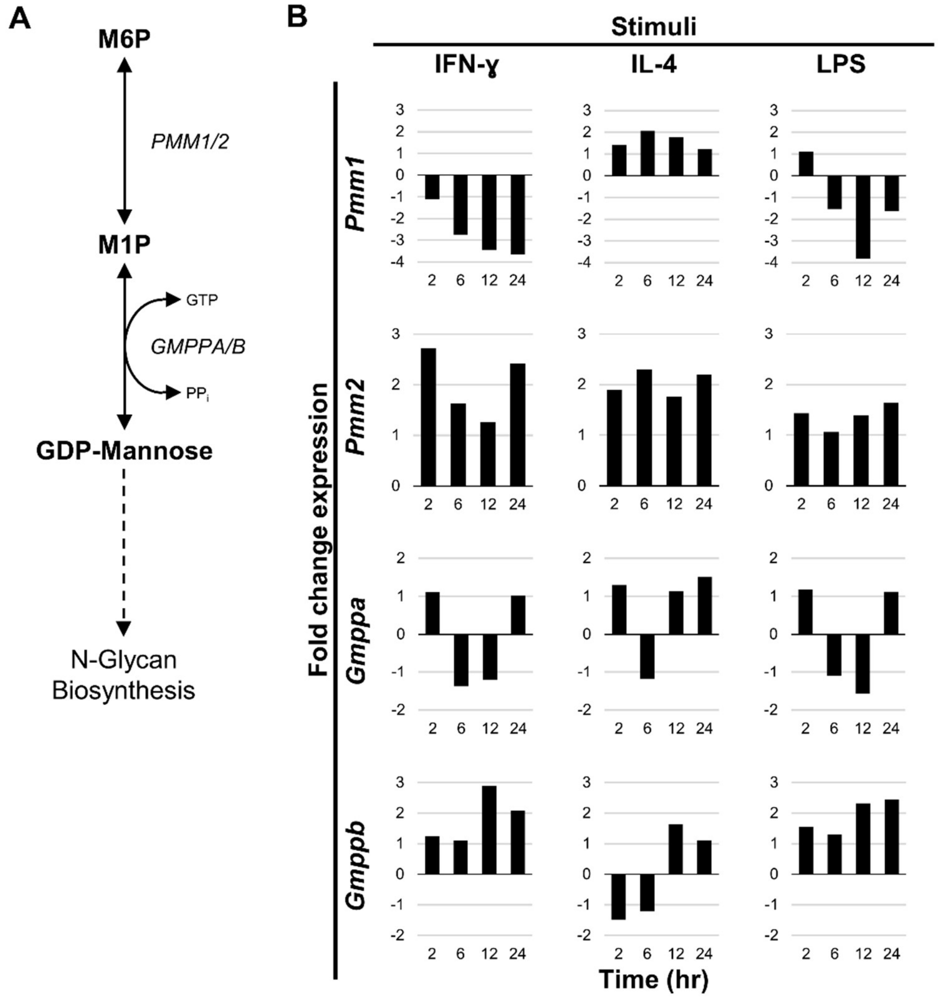

Figure S3. Metabolism of mannose for N-glycan biosynthesis **(A)** and fold change expression of *Gmppa*, *Gmppb*, *Pmm1*, and *Pmm2* in bone marrow-derived murine MΦs upon differential stimuli **(B)**. Data has been derived from Zhang *et al.* to generate these plots [39]. Abbreviations denote: GDP, guanosine diphosphate; *Gmppa*, GDP-mannose pyrophosphorylase A; *Gmppb*, GDP-mannose pyrophosphorylase B; GTP, guanosine triphosphate; IFN-γ, interferon-γ; IL-4, interleukin-4; LPS, lipopolysaccharide; M1P, mannose 1-phosphate; M6P, mannose 6-phosphate; *Pmm1*, phosphomannomutase 1; *Pmm2*, phosphomannomutase 2.

Amanda L. Fuchs *et al.*, Figure S4

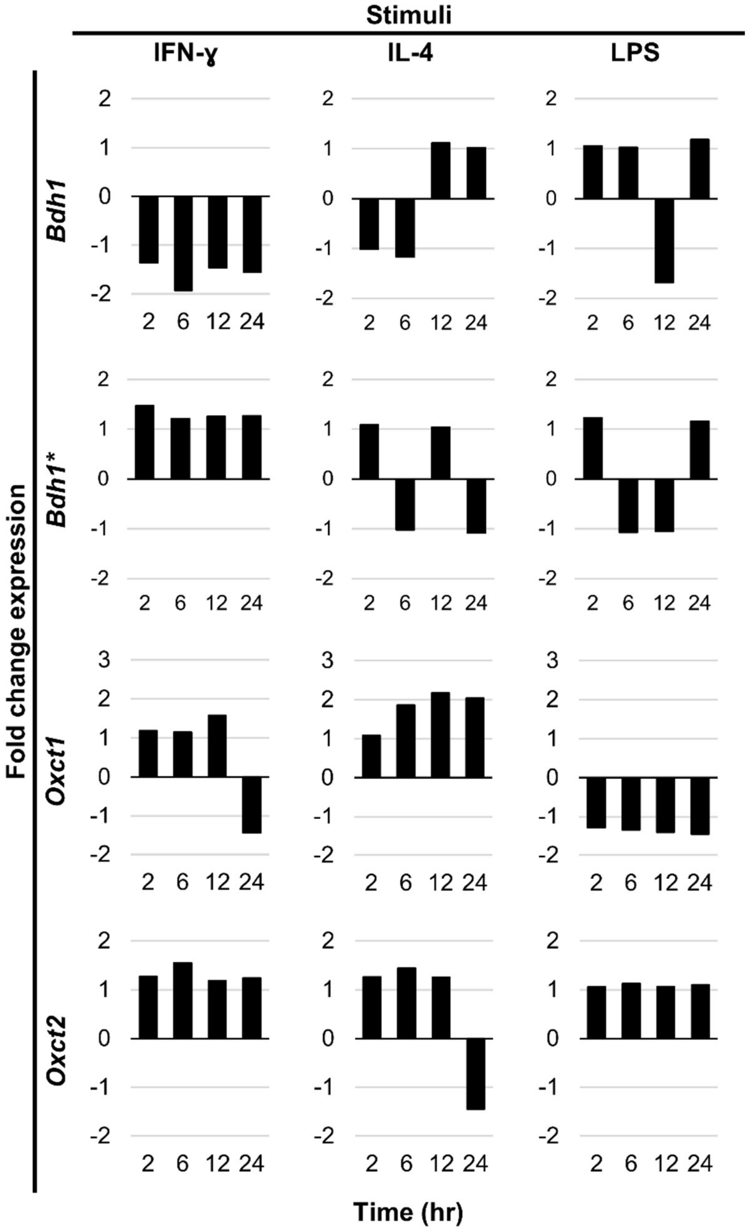

Figure S4. Fold change expression of *Bdh1* (mMR028403), *Bdh1** (mMC011803), *Oxct1*, and *Oxct2* in bone marrow-derived murine MΦs upon differential stimuli. Data has been derived from Zhang *et al*. to generate these plots [39]. Abbreviations denote: *Bdh1*, 3-hydroxybutyrate dehydrogenase 1; IFN-γ, interferon-γ; IL-4, interleukin-4; LPS, lipopolysaccharide; *Oxct1/2*, 3-oxoacid Co-A transferase 1/2.

Amanda L. Fuchs *et al.*, Figure S5

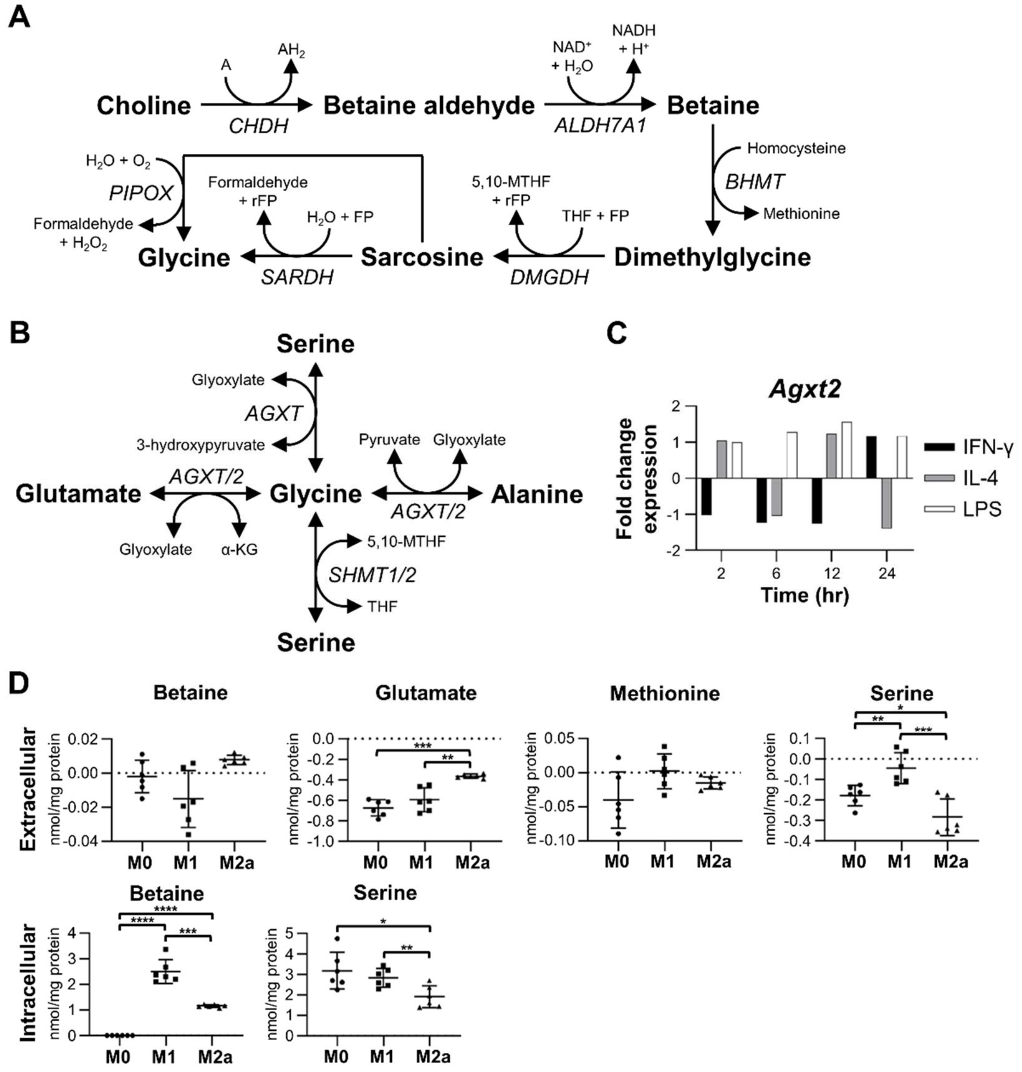

Figure S5. Generation of glycine from **(A)** choline or **(B)** glutamate, serine, and alanine. **(C)** Fold change expression of *Agxt2* in bone marrow-derived murine MΦs upon differential stimuli. Data has been derived from Zhang *et al.* to generate this plot [39]. **(D)** Quantitative levels of corresponding metabolites detected in intra- and extracellular MΦ metabolite extracts (mean ± SD). Statistical significance (p) was measured using two-tailed unpaired parametric t-tests with Welch's correction, whereby *, $p < 0.05$; **, $p < 0.01$; ***, $p < 0.001$; ****, $p < 0.0001$. Abbreviations denote: 5,10-MTHF, 5,10-methylenetetrahydrofolate; α-KG, α-ketoglutarate; A, acceptor; AH_2, reduced acceptor; *ALDH7A1*, betaine aldehyde dehydrogenase; *AGXT*, alanine-glyoxylate aminotransferase; *BHMT*, betaine-homocysteine S-methyltransferase; *CHDH*, choline dehydrogenase; *DMGDH*, dimethylglycine dehydrogenase; FP, electron-transfer flavoprotein; IFN-γ, interferon-γ; IL-4, interleukin-4; LPS, lipopolysaccharide; NAD^+, nicotinamide adenine dinucleotide; NADH, reduced nicotinamide

adenine dinucleotide; *PIPOX*, sarcosine oxidase; rFP, reduced electron-transfer flavoprotein; *SARDH*, sarcosine dehydrogenase; *SHMT*, serine hydroxymethyltransferase; THF, tetrahydrofolate.

Amanda L. Fuchs *et al.*, Figure S6

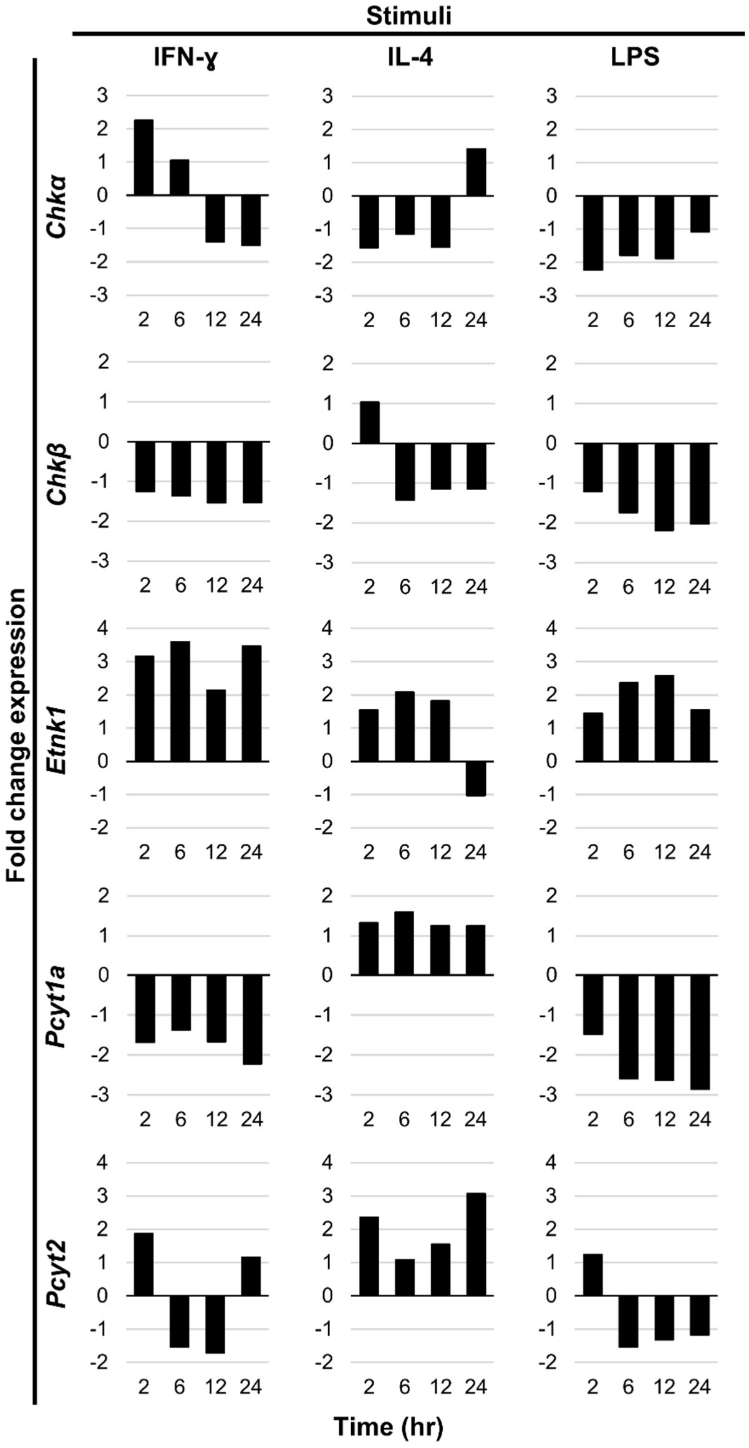

Figure S6. Fold change expression of *Chkα*, *Chkβ*, *Etnk1*, *Pcyt1a*, and *Pcyt2* in bone marrow-derived murine MΦs upon differential stimuli. Data has been derived from Zhang *et al.* to generate these plots [39]. Abbreviations denote: *Chkα*, choline kinase α; *Chkβ*, choline kinase β; *Etnk1*, ethanolamine kinase 1; IFN-γ, interferon-γ; IL-4, interleukin-4; LPS, lipopolysaccharide; *Pcyt1a*, phosphate cytidylyltransferase 1α; *Pcyt2*, phosphate cytidylyltransferase 2.

Amanda L. Fuchs *et al.*, Figure S7

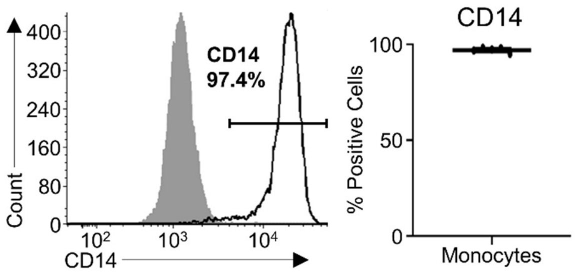

Figure S7. Purity of isolated monocytes. Representative histogram of purified CD14⁺ monocytes (left) and pooled data from 5 independent experiments, mean ± SEM (right).

Amanda L. Fuchs *et al.*, Figure S8

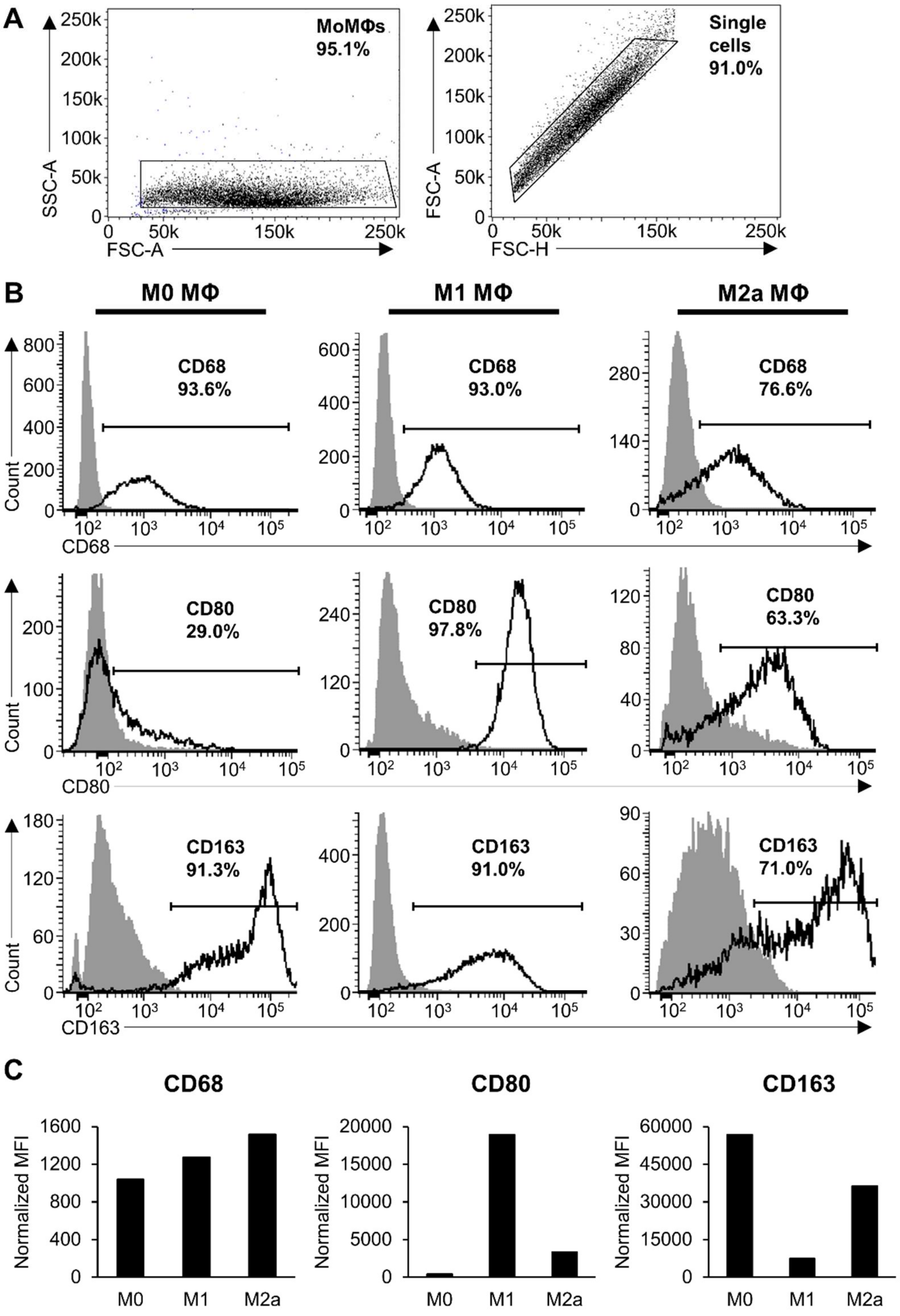

Figure S8. Phenotype of primary human monocyte-derived macrophages (MoMΦs). **(A)** Gating strategy for FACS analysis of MoMΦs; **(B)** Dot plots of CD68, CD80, and CD163 expression by M0, M1, and M2a MoMΦs; **(C)** Normalized mean fluorescence intensity (MFI) of CD68, CD80, and CD163 expression by M0, M1, and M2a MoMΦs.

Amanda L. Fuchs *et al.*, Table S1

Table S1. 1D ^{1}H NMR intra- and extracellular metabolite limit of detection (LOD) values.[1]

Extract	Metabolite	LOD (µM)
Intracellular	Arginine	3
	ATP	1
	Betaine	1
	Glucose-1-phosphate	1
	NAD^+	0.5
	Niacinamide	1
	Quinolinate	1
Extracellular	Fumarate	1

[1] LOD values were established by evaluation of signal to noise ratios in our experimental 1D ^{1}H NMR spectra, Chenomx NMR Suite software, and its accompanying Chenomx 600 MHz metabolite library. Abbreviations denote: ATP, adenosine triphosphate; NAD^+, nicotinamide adenine dinucleotide.

Amanda L. Fuchs *et al.*, Table S2

Table S2. Discriminatory metabolites in intracellular extracts associated with M1 vs. M2a MΦ activation.[1]

**Metabolite**	**M1 vs. M2a MΦs**	
	FC	*p*-value
Acetate	1.27	**
ADP	-1.36	***
AMP	-1.83	****
Aspartate	-1.32	**
ATP	9.14[2]	****
Betaine	2.16	***
Choline	1.91	****
Creatine	-1.46	*
Creatine phosphate	1.32	***
Fumarate	-1.86	****
Glucose	-1.47	*
Glutamate	-2.19	***
Glutamine	1.36	**
GSH	-1.34	**
GTP	-1.49	***
myo-Inositol	-4.57	****
NAD^+	-4.06	****
NADPH	-3.20	****
Niacinamide	5.19[2]	****
O-phosphocholine	-9.51	***
O-phosphoethanolamine	2.03	***
Proline	-1.39	*
Propionate	1.39	**
Quinolinate	25.90[2]	****
Serine	1.49	**
Succinate	1.48	*
Taurine	1.45	***
UMP	-1.61	****
Valine	1.18	*

[1] Metabolites were selected based upon fold change (FC) and statistical significance of intracellular metabolite concentrations between M1 and M2a MΦs; (nmol/mg protein; calculated from metabolite spectral fitting using the Chenomx NMR Suite software and the standard Chenomx 600 MHz metabolite library). Fold changes were calculated

relative to M2a MΦs, whereby increases are shown as positive values and decreases are shown as negative values. Statistical significance (p) was measured using two-tailed unpaired parametric t-tests with Welch's correction, whereby *, $p < 0.05$; **, $p < 0.01$; ***, $p < 0.001$; ****, $p < 0.0001$. Abbreviations denote: ADP, adenosine diphosphate; AMP, adenosine monophosphate; ATP, adenosine triphosphate; GSH, reduced glutathione; GTP, guanosine triphosphate; NAD^+, nicotinamide adenine dinucleotide; NADPH, reduced nicotinamide adenine dinucleotide phosphate; UMP, uridine monophosphate. [2] Fold change (FC) was calculated using limit of detection (LOD) values (see Table S1) for MΦ activation state in which a given metabolite was not detected.

Amanda L. Fuchs *et al.*, Table S3

Table S3. Relative fold change expression of select genes in bone marrow-derived murine MΦs upon differential stimuli.[1]

Gene	Stimuli	Time (hr)			
		2	6	12	24
Agxt2	IFN-γ	-1.03	-1.24	-1.27	1.18
	IL-4	1.05	-1.05	1.23	-1.39
	LPS	1.01	1.28	1.56	1.17
Bdh1	IFN-γ	-1.37	-1.93	-1.47	-1.56
	IL-4	-1.01	-1.17	1.12	1.02
	LPS	1.06	1.03	-1.68	1.18
*Bdh1**	IFN-γ	1.47	1.21	1.25	1.27
	IL-4	1.08	-1.02	1.04	-1.08
	LPS	1.23	-1.07	-1.05	1.16
Chkα	IFN-γ	2.25	1.05	-1.39	-1.49
	IL-4	-1.55	-1.14	-1.54	1.42
	LPS	-2.22	-1.78	-1.88	-1.08
Chkβ	IFN-γ	-1.25	-1.37	-1.54	-1.54
	IL-4	1.04	-1.43	-1.15	-1.15
	LPS	-1.22	-1.74	-2.19	-2.03
Etnk1	IFN-γ	3.16	3.60	2.13	3.47
	IL-4	1.55	2.08	1.82	-1.03
	LPS	1.44	2.37	2.58	1.55
Gmppa	IFN-γ	1.12	-1.37	-1.20	1.01
	IL-4	1.30	-1.18	1.14	1.51
	LPS	1.19	-1.10	-1.57	1.12
Gmppb	IFN-γ	1.24	1.10	2.89	2.09
	IL-4	-1.48	-1.20	1.64	1.11
	LPS	1.55	1.31	2.32	2.44
Oxct1	IFN-γ	1.19	1.15	1.58	-1.44
	IL-4	1.08	1.86	2.17	2.03
	LPS	-1.29	-1.35	-1.41	-1.46
Oxct2	IFN-γ	1.27	1.55	1.18	1.24
	IL-4	1.26	1.44	1.25	-1.45
	LPS	1.05	1.13	1.06	1.10
Pcyt1a	IFN-γ	-1.68	-1.37	-1.66	-2.22
	IL-4	1.33	1.59	1.26	1.24
	LPS	-1.47	-2.59	-2.63	-2.85
Pcyt2	IFN-γ	1.87	-1.54	-1.71	1.15
	IL-4	2.36	1.08	1.55	3.07
	LPS	1.24	-1.54	-1.31	-1.17
Pmm1	IFN-γ	-1.12	-2.74	-3.45	-3.66
	IL-4	1.40	2.06	1.77	1.22
	LPS	1.11	-1.54	-3.83	-1.63
Pmm2	IFN-γ	2.71	1.63	1.27	2.41
	IL-4	1.90	2.29	1.76	2.20
	LPS	1.43	1.07	1.39	1.63

[1] Fold changes were calculated using supplementary microarray data for *Agxt2*, *Bdh1*, *Bdh1**, *Chkα*, *Chkβ*, *Etnk1*, *Gmppa*, *Gmppb*, *Oxct1*, *Oxct2*, *Pcyt1a*, *Pcyt2*, *Pmm1*, and *Pmm2* genes, with UNIQIDs of mMA035026, mMR028403, mMC011803, mMC011615, mMA034846, mMR029849, mMR028432, mMC009282, mMC013095, mMC019363, mMC002638, mMC007591, mMC004009, and mMA034923, respectively, relative to control MΦs (data derived from Zhang *et al*.) [39]. Increases are shown as positive values and decreases are shown as negative values. Abbreviations denote: *Agxt2*, alanine-glyoxylate aminotransferase 2; *Bdh1*, 3-hydroxybutyrate dehydrogenase 1; *Chkα*, choline kinase α; *Chkβ*, choline kinase β; *Etnk1*, ethanolamine kinase 1; *Gmppa*, GDP-mannose pyrophosphorylase A; *Gmppb*, GDP-mannose pyrophosphorylase B; IFN-γ, interferon-γ; IL-4, interleukin-4; LPS, lipopolysaccharide; *Ocxt1/2*, 3-oxoacid Co-A transferase 1/2; *Pcyt1a*, phosphate cytidylyltransferase 1α; *Pcyt2*, phosphate cytidylyltransferase 2; *Pmm1*, phosphomannomutase 1; *Pmm2*, phosphomannomutase 2.
